# Four Centuries of Change in Northeastern United States Forests

**DOI:** 10.1371/journal.pone.0072540

**Published:** 2013-09-04

**Authors:** Jonathan R. Thompson, Dunbar N. Carpenter, Charles V. Cogbill, David R. Foster

**Affiliations:** 1 Smithsonian Conservation Biology Institute, Smithsonian Institution Front Royal, Virginia, United States of America; 2 Department of Forest and Wildlife Ecology, University of Wisconsin, Madison, Wisconsin, United States of America; 3 Harvard Forest, Harvard University, Petersham, Massachusetts, United States of America; DOE Pacific Northwest National Laboratory, United States of America

## Abstract

The northeastern United States is a predominately-forested region that, like most of the eastern U.S., has undergone a 400-year history of intense logging, land clearance for agriculture, and natural reforestation. This setting affords the opportunity to address a major ecological question: How similar are today's forests to those existing prior to European colonization? Working throughout a nine-state region spanning Maine to Pennsylvania, we assembled a comprehensive database of archival land-survey records describing the forests at the time of European colonization. We compared these records to modern forest inventory data and described: (1) the magnitude and attributes of forest compositional change, (2) the geography of change, and (3) the relationships between change and environmental factors and historical land use. We found that with few exceptions, notably the American chestnut, the same taxa that made up the pre-colonial forest still comprise the forest today, despite ample opportunities for species invasion and loss. Nonetheless, there have been dramatic shifts in the relative abundance of forest taxa. The magnitude of change is spatially clustered at local scales (<125 km) but exhibits little evidence of regional-scale gradients. Compositional change is most strongly associated with the historical extent of agricultural clearing. Throughout the region, there has been a broad ecological shift away from late successional taxa, such as beech and hemlock, in favor of early- and mid-successional taxa, such as red maple and poplar. Additionally, the modern forest composition is more homogeneous and less coupled to local climatic controls.

## Introduction

The land use history of the northeastern United States is well documented but its ecological consequences remain poorly understood, especially at a regional scale [1]–[4]. For more than 10,000 years native people cleared modest areas along waterways and seasonal settlements and managed some upland areas through sporadic understory burning [5]. Even so, the region was overwhelmingly forested and chiefly governed by non-anthropogenic disturbances and successional dynamics until around 1650, when two centuries of logging and agricultural clearing were initiated that removed more than half of the forest cover and cut over almost all of the rest. Outside of the far north and rugged mountainous regions, the northeast became a predominantly humanized agrarian landscape. Forest cover reached its nadir in the mid nineteenth century, after whichagricultural expansion to the Midwest and eastern industrialization resulted in widespread farm abandonment, population concentration and, in turn, a century of natural reforestation and forest growth [6],[7]. The emerging forest supported new wood-based industries and natural processes including forest succession interrupted by damage from severe storms such as the powerful 1938 Hurricane, which was compounded by subsequent salvage logging on massive scales [6], [8], [9]. The modern landscape appears to have recently reached its apex of reforestation and the region is again experiencing a net loss of forest cover. While agricultural land cover continues to decline throughout the region, land cover transitions to developed uses now override reforestation [10]. As of 2010, approximately 80 percent of the region was forested, though less than one-percent of old-growth forest remains intact [11].

Given the long and tumultuous history of landscape change, an important ecological question emerges: How similar is the composition of today's forests compared to those existing prior to European colonization? This question has relevance far beyond the northeastern U.S. Conversion of natural ecosystems for agriculture and developed uses are a hallmark of human civilization. Indeed, more than one-third of the land surface of the earth is currently under agricultural use and more than half of the human population lives in urban regions [12]. Throughout human history shifts in economic forces have resulted in land abandonment followed by natural reforestation, as happened in the northeastern U.S. [13], in Central America after the Mayan collapse [14], in parts of the Ecuadorian Amazon [15] and western Europe, and elsewhere [16]. Large-scale Reforestation and natural restoration of forest regions are also major goals for modern conservation. Therefore the question looms: how do regional ecosystems recover from such massive scales of anthropogenic disturbance? Are the secondary ecosystems qualitatively different in terms of composition, function, and services? Or, does an inherent resilience and species fidelity to environmental conditions drive regional ecosystems back to their pre-disturbance condition? Because the processes of regional disturbance and recovery occur over such large spatial and long temporal scales, these questions are rarely addressed empirically.

Here we use a unique dataset, singular in its geographic scope, to quantify the Northeast U.S. region's response to long-term, broad-scale shifts in land use. We compare modern forest data to historical vegetation data gleaned from archival, colonial land-survey notes collected over a nine-state area. Colonial surveyors described the details of “witness trees,” which served as semi-permanent monuments of survey corners. Ecologists have been using these inadvertent forest inventories to reconstruct pre-colonial forest composition for almost a century (e.g., [17]). The records contain a wealth of information about North America's historical ecosystems [18]. But because the records were not designed to record vegetation, they do have several limitations and biases, (see [19], [20] for reviews). In the Northeast, the witness trees demarcated the corners of lots ranging in size from 0.5 to 65 hectares [2],[21]. Unlike the more systematic approach used by the General Land Office, concurrent with westward expansion from Ohio to California, witness tree data in the Northeast were collected using a variety of methods, typically do not include tree size, and commonly identify trees only to genera as opposed to species [19]. Despite these comparative shortcomings, ecologists have made great use of town proprietor records and the references to witness trees therein.

Witness tree have been used extensively throughout the northeast to reconstruct historical forest composition, without making comparisons to modern forest conditions [21]–[27]. Far fewer studies have quantified compositional change and these have all focused on relatively small regions. Their localized perspective limits our understanding of the regional variability and the relative importance of land use history and biophysical setting for determining the degree and nature of forest change. A review of these studies shows some consistent changes. For instance, the loss of American chestnut (*Castanea dentata*) due to an introduced fungal blight (*Cryphonectaria parasitica*) is evident throughout the region [28]. But looking across these studies shows that changes in composition have not been uniform throughout the Northeast. Of course, there are a number of reasons to expect different patterns in different places related to variation in: land use, edaphic factors, disturbance regimes or position relative to climatic drivers and floristic boundaries. For example, Bürgi et al. [29] compared witness trees to modern inventory data in two counties in Massachusetts and two in Pennsylvania and found an almost two-fold difference in the degree of overall compositional change between study areas. Similarly, some studies suggest that the modern forest is compositionally more homogeneous than the forests they replaced [4], [30] while others have found the opposite [31]. Trends in the abundance of individual taxa also vary widely. The abundance of oak, for example, has increased in some areas [32] and decreased in others [33]. Variation in land use is often assumed to be a primary determinant of change and, indeed, agricultural clearing, harvesting bark for tanning, and logging have been correlated with compositional change [29], [31], [34].

From these local-scale studies we know that the forests have changed, but we are unable to put these changes in the context of the massive shifts in land-use that reshaped the regional ecosystem during the past 400 years. By assembling a comprehensive geo-database of witness-tree records in the Northeast, including more than 150,000 tree records that have not been published before, we addressed several significant questions: (1) How does the magnitude of compositional change vary across the region? (2) Do changes in composition reflect the region's land-use history mosaic and environmental gradients? (3) Is the regional forest recovery producing a landscape that is compositionally more or less diverse to what existed prior to colonization? (4) Is the nature of compositional change consistent across the region? (5) What are the patterns of change among individual taxa? By answering these questions we begin to quantify the resilience of regional ecosystems subjected to large-scale shifts in land use.

## Methods

### Study Region

**Figure 1:**
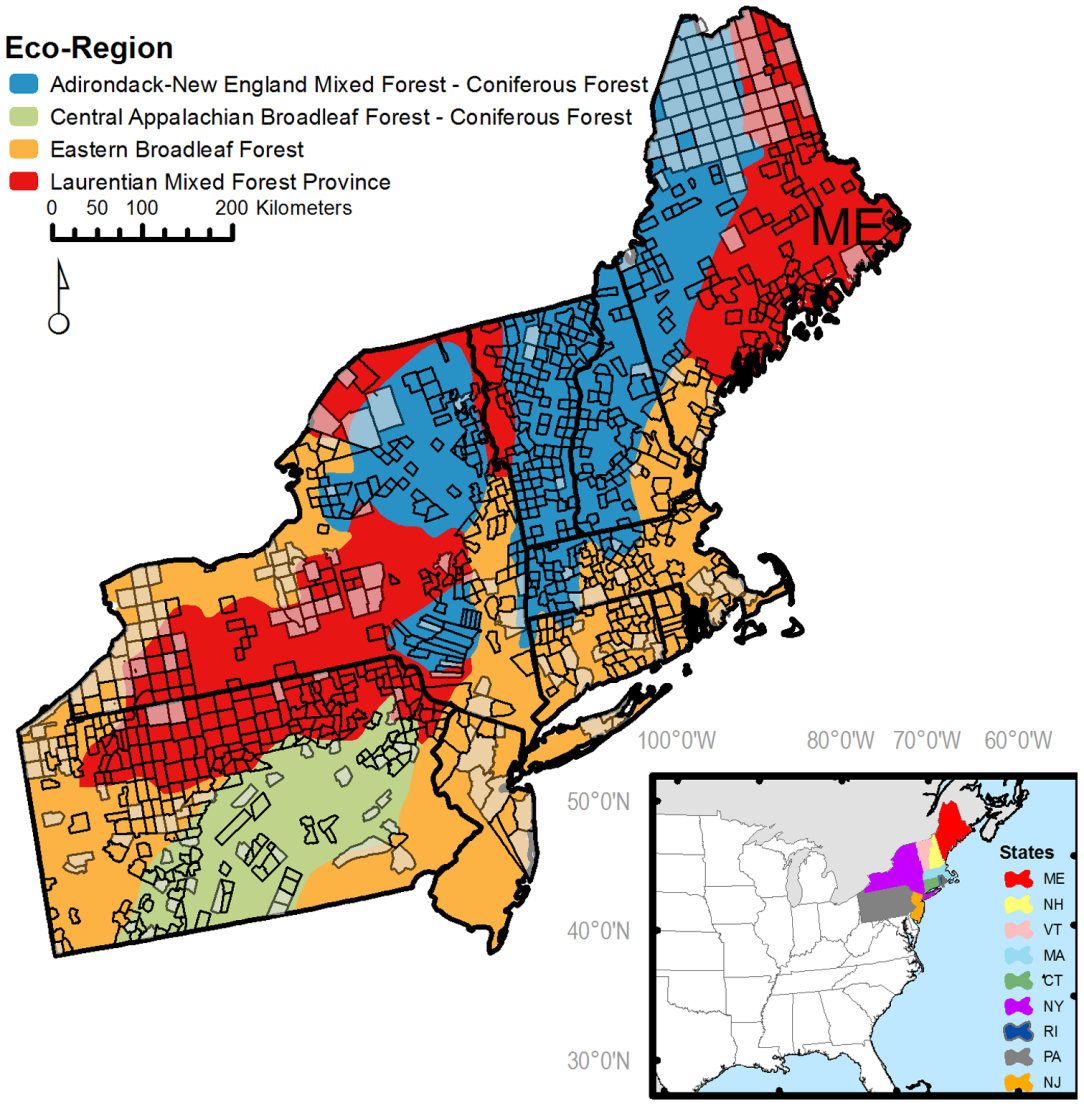
The nine-state study region in the northeastern United States. Colors correspond to U.S. Forest Service designated ecoregions. The inner polygons correspond to the 1280 colonial town where pre-colonial forest data were collected. Of these, 701 contained an adequate sample of witness trees and modern forest data to permit comparative analyses. Town with insufficient data are grayed-out in the map. Inset: The location of the study area within the conterminous United States.

We examined changes in forest composition within a sample of colonial townships distributed throughout a nine state region of the northeastern U.S. (Figure 1), ranging latitudinally from northern Maine (47°30′ N) to southern New Jersey (39°30′ N) and longitudinally from western Pennsylvania (67° W) to the Atlantic Ocean (80°30′ W). The study area encompasses 4.33×10^5^ km^2^ and spans nine physiographic provinces, primarily in the Appalachian Highlands Division, but also extending into portions of the Atlantic and Interior Plains. The region's rolling topography is interrupted by several discrete sub-mountain ranges belonging to the greater Appalachian Chain. Several major rivers including the Hudson, Connecticut, Merrimack, Susquehanna, and Penobscot form low-elevation valleys across the study area. Much of the present geology of the region was shaped by the last glaciation (c. 20,000 ybp). The region is influenced by a range of climatic conditions; annual mean temperatures range from 3 to10°C (mean Jan temp  = −6°C; mean July temp  = 19°C), and average annual precipitation ranges from 79 to 255 cm. The study area includes four USFS designated ecoregions (Figure 1), defined based on a broad set of ecologically-relevant attributes [35], which we used to stratify our samples.

### Pre-colonial data

We used the relative abundance of witness tree taxa (i.e., proportion of each taxon) identified in Table 1 within proprietary towns, as our metric of pre-colonial forest composition (c.f. [21]). Proprietary towns (hereafter “towns”) were granted by the colonies and states to absentee individuals to encourage colonization and “improvement” of the land throughout the period spanning from just after English colonization (1620) to after the creation of the Erie Canal (1825). Towns were usually 6-miles square (≈100 km^2^) and regularly shaped. Within each town, individual lots were established and surveyed using witness trees (WT) as markers. The original sources of the WT data typically include proprietors' records, field books, manuscripts, maps and published records of town land surveys before colonization [21]. Town lotting surveys are the authoritative source, but when unavailable or inadequate other sources containing contemporary tree data were used. The witness trees are thus a relatively objective sample of forest composition prior to colonization. The land surveyors used English colloquial names to describe the trees and while they were skilled naturalists they often did not discern individual species within a genus. To reduce taxonomic uncertainty and ensure consistency across surveys we classified all trees into widely represented genera, following Cogbill et al. [21]. While this introduces some species ambiguity into the groupings, it is unavoidable, since surveyors of pre-colonial witness trees often did not distinguish species within genera. We assembled available town land survey records within the region, totaling 1280 towns and 325,000 trees. WT data are publicly available from individual town halls and archives throughout the region. Approximately 55 percent of the WT towns had been utilized in previous published studies, each of which focused on a smaller region.

**Table 1:** Taxa groupings with stem counts and occurrences within 701 colonial towns that met the minimum threshold to be included in the analysis.

Taxon	Pre-Colonial		Modern	
	Towns	Stems	Towns	Stems
Ashes	547	3916	500	3996
Basswood	390	1655	180	538
Beech	615	31909	474	7525
Birches	655	9678	657	11467
Blackgum	118	460	94	475
Cedar	132	976	116	2278
Cherries	220	546	462	4565
Chestnut	332	7616	11	11
Cypress	23	82	4	34
Elms	331	1433	152	555
Fir	216	2047	222	6178
Hemlock	605	15817	467	8184
Hickories	309	7493	208	1094
Hornbeam	384	1683	245	1017
Magnolias	63	162	46	108
Maple	692	17017	699	33167
Oak	489	49014	467	11662
Pines	516	12176	442	7398
Poplars	258	1109	345	2401
Spruces	355	9288	296	5076
Sycamore	84	162	4	10
Tamarack	63	181	39	269
Tulip	113	335	73	368
Walnuts	156	421	27	69
TOTAL	701	176715	701	109784

### Modern Data

The modern tree data come from the USDA Forest Service Forest Inventory and Analysis (FIA) program. We used the FIA census, spanning 2003–2008. FIA plots were sampled at an intensity of one plot per 2400-ha throughout the study region. Each plot consists of four, 7.3-m fixed-radius subplots (totaling 168-m^2^), on which all trees >1.3-m in height are identified to species and the dbh recorded. . FIA protocols and data are publically available online (http://apps.fs.fed.us/fiadb-downloads/datamart.html). However, we obtained coordinates for the inventory plots, which allowed us to pair FIA plots with the WT town in which they reside, from the US Forest Service pursuant to a Memorandum of Understanding #09MU11242305123 between the U.S. Forest Service and Harvard University. We excluded FIA plots that were not classed as “Forest” within the FIA Condition table or contained <10 trees >12.5-cm dbh. In the remaining plots we excluded all trees <12.5-cm dbh to reduce the potential for bias against smaller trees within the pre-colonial data [29], [36]. (Based on the findings of Wang et al [37], we explored a 20-cm dbh threshold for inclusion. This resulted in a large reduction in number of towns meeting the sample intensity criteria outlined below, with little qualitative change in our findings.) We excluded any towns with <2 qualifying FIA plots. We then binned the tree species into the same 20 taxa used for the WTs (Table 1) and calculated the relative abundance in each plot.

### Assessing the sample intensity

The density of WTs and FIA plots within towns varied widely. We used an approach somewhat akin to rarefaction analysis (e.g. [38]) to estimate the minimum density of FIA plots and WTs at which tree compositional diversity had been adequately sampled – i.e. to determine whether or not each town had sufficient tree data to include in our analyses. Our approach relied on the fact that tree diversity increases asymptotically with the addition of each new WT or FIA plot. By using bootstrap sampling and fitting Michalis-Menton (M-M) functions, we estimated the density of WTs and FIA plots at which the full complement of diversity was represented. This density was used as a minimum threshold for determining whether or not to include a town in the analyses. 

**Figure 2:**
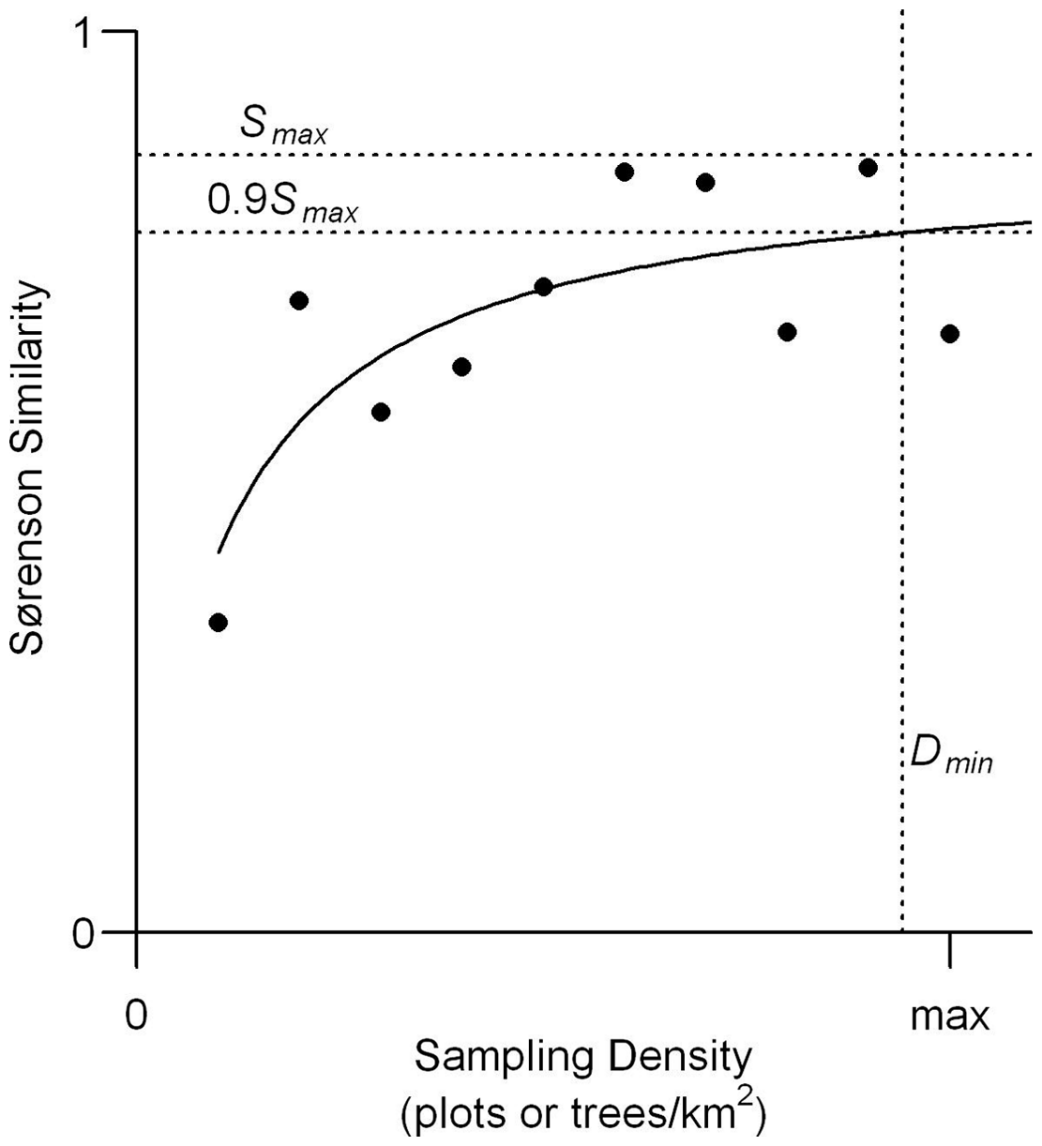
An illustrative example of one bootstrap sample of FIA plots or witness trees used to estimate the minimum sampling intensity necessary to capture the forest compositional diversity of a town. Each dot represents the Sørensen similarity between a bootstrap sample of FIA plots or bins of witness trees, and the town's composition with all plots included, for n in one through the total number of plots/bins in the town. The curve is a Michaelis-Menten function fit to the similarity values. S_max_ is the curve's asymptote and D_min_ is the plot or tree density at 0.9S_max_. D_min_ was averaged over 100 sets of bootstrap samples from each of the most plot-dense towns in an ecoregion to determine the ecoregion's minimum sampling density.

More specifically, our procedure for determining adequate FIA plot density was as follows: (1) We stratified the study region by ecoregions (Figure 1). (2) Within each ecoregion, we selected the most plot-dense towns, taking only those towns with ≥5 FIA plots and that were within the upper tercile of plot density (plots/km^2^). (3) For each town within this subset, we iteratively took 100 sets of bootstrapped samples, with each set consisting of a sample of one FIA plot, a sample of two FIA plots (sampled with replacement), and so on up to the total number of FIA plots in that town. (4) For each bootstrap sample within a set we calculated the mean Sørenson distance (see below) between the sample's relative composition and the relative composition with all plots included. As the number of plots sampled increases, Sørenson similarity tends to initially increase sharply before slowly leveling off as composition stabilizes (Figure 2). (5) Accordingly, for each set of bootstrap samples we fit an M-M curve and recorded the asymptote, *S_max_ S_max_* is an estimate of the similarity between two random samples of the town's forest when plot density is impossibly large and is typically slightly less than one, or complete similarity, due to variation introduced by bootstrap sampling. (6) We calculated a threshold plot density, *D_min_,* as the minimum plot density required to reach a given proportion of *S_max_.* Since reaching *S_max_* would require infinite plot density, we decided that the plot density required to reach 90 percent of *S_max_* would be adequate to approximate a town's true forest composition. (7) We averaged *D_min_* over all 100 sample sets from each town, and then over all towns within each ecoregion. This ecoregion grand mean, *D_min_*, was taken to be the ecoregion-wide threshold plot density necessary to capture a town's compositional diversity.

We followed a similar procedure for determining the adequate number of WTs necessary to capture the compositional diversity within a town, except that we iteratively sampled bins of 20 trees, as opposed to FIA plots, before fitting the M-M function. We also used only those towns with at least 100 WTs and that were within the upper tercile of tree density (WTs per km^2^).

### Comparing pre-colonial to modern forest composition

We first compared the relative abundance of each taxon across the study region and within each ecoregion. We used paired Monte Carlo tests (10,000 randomizations of group membership) to compute p-values describing the probability of encountering differences in relative abundance that were at least as large as those observed from chance alone, given the distribution of data [39].

We calculated the Sørensen's distance measure for several analyses of compositional change, both in time and in space. Sørensen is defined as:
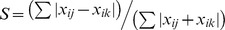
Where *x_ij_* is the abundance of taxon *i* in town *j* and *x_ik_* is the abundance of taxon *i* in town *k*. Values of *S* range from 0, indicating identical composition, to 1 for no overlap in composition. Empirical analyses have shown Sørensen's distance to be a robust measure of compositional dissimilarity in ecological data because it retains sensitivity in more heterogeneous data sets and gives less weight to outliers [40].

For each town we calculated the Sørensen's distance from its pre-colonial composition to its modern composition to estimate the degree of change over time. We mapped the change in community composition and compared differences between the ecoregions using a permutation test to compute p-values describing the probability of encountering differences in dissimilarity that were at least as large as those observed from chance alone [39]. To understand whether the degree of community change over time (i.e., Sørensen's) was spatially structured, we constructed a Moran's I spatial correlogram. We experimented with several distance classes within the correlogram; based on the distribution of town-to-town distances and the average nearest neighbor distance (≈7.4 km based on town centroids), we settled on 15 km uniform distance classes. The statistical significance of spatial autocorrelation within each distance class was calculated using a permutation test with α = 0.05.

We ordinated the pre-colonial and modern towns in forest community space to visually examine changes in community composition. We constructed a non-metric multidimensional scaling (NMDS) ordination (50 random starts; scaling; centering; PC rotation; half-change scaling [41]) using the “MetaMDS” function in the Vegan v2.0-5 library in the R statistical language [42]. Wisconsin double standardization was applied and the Sørensen's measure was used. The appropriate number of dimensions (axes) was determined by plotting final stress values against the number of dimensions on a scree plot.

We used several approaches to examine potential evidence of compositional homogenization and changes in the strength of associations between community structure and the environment. First, following Rooney et al. [43], we calculated the average dissimilarity (i.e., Sørensen's) of each town to all other towns within the study area and within each ecoregion within at each time period. We then compared the mean town-to-town dissimilarity between the pre-colonial and modern data using a permutation test. A reduction in town-to-town dissimilarity over time is evidence that the forests have converged in terms of compositional diversity. To gauge changes in the spatial structure of forest community composition between the time periods, we constructed Mantel correlograms [44] using the “mantel.correlog” function within the Vegan v 2.0-5Library of the R statistical language [42]. In brief, the Mantel's test of matrix correlation, *r_M,_* is calculated at multiple distance classes and plotted across the full range of distances. In those distance classes where *r_M_* is positive and significantly different than zero, the multivariate similarity among towns is higher than expected by chance. Conversely, when *r_M_* is negative and significantly different than zero, then the towns are more dissimilar than expected by chance. Whether the correlation is significantly different than zero is determine through a permutation test with a Bonferoni corrected α of 0.05. To understand if the strength of associations between climatic and edaphic variables has changed over time, we used Mantel correlation tests between the community distance matrices from both time periods and temperature (GDD), average precipitation, average elevation, and percent sand (as described in Table 2). Note that *r_M_* are not equivalent to the more familiar Pearson's *r* and should not be directly compared [45].

**Table 2:** Land use and bio-physical predictor variables.

Variable	Explanation/Source
Temperature	Mean annual growing degree days using a 0°C base [73].
Precipitation	Mean annual precipitation in millimeters within each town [73].
Elevation	Average elevation above sea level in meters within each town calculated using a 30 meter resolution digital elevation model.
Ruggedness	Standard deviation of elevation in meters within the town calculated using a 30 meter resolution digital elevation model.
Peak agricultural land cover	Maximum proportion of land in agriculture between 1850 and 1997 according to county-level census and agricultural survey data [74].
Peak agriculture year	Year during which the maximum proportion of a counties land was in agriculture according to county-level census and agricultural survey data from 1850 to 1997 [74].
Rate of agricultural decline	Proportion of land taken out of agriculture each year, according to a regression of the proportion of agricultural land (PAL) beginning in the year of peak agriculture and ending when PAL went below 10% or came within 20% of its minimum (i.e., when decline flattens out). Based on county-level census and agricultural survey data [74].
Canopy cover	Density of tree canopy (as a percentage of the area of the town) based on the Multi-Resolution Land Characteristics consortium's 2001 National Landcover Database [75].
Soil sand	Percentage of sand in the mineral portion of the surface layer of the soil calculated as a weighted area of the town using the U.S. General Soil Map STATSGO2 [76].
Soil clay	Percentage of clay in the mineral portion of the surface layer of the soil calculated as a weighted area of the town using the U.S. General Soil Map STATSGO2 [76].
Latitude	At centroid of the town.
Longitude	At centroid of the town.

Finally, to evaluate the relationships between compositional change and the suite of predictor variables identified in Table 2 further, we used regression tree analysis (RTA) with the Sørenson's distance between time periods as the response variable. RTA is a non-parametric technique that recursively partitions a dataset into subsets that are increasingly homogeneous with regard to the response [46]. We used an implementation of RTA, called conditional inference trees using the “ctree” function in the PARTYv 1.0–7 library [47] within the R statistical language [42]. Conditional inference trees establish partitions based on the lowest statistically significant P-value that is obtainable across all levels of all predictor variables, as determined from a Monte Carlo randomization test. This minimizes bias and prevents over-fitting and the need for pruning [48].

## Results

Of 1280 towns in the study area with witness tree data, 904 contained two or more forested FIA plots (Figure 1). Of these, 761 towns contained sufficient WT density to meet the sampling density threshold. Most towns with insufficient WT density were clustered in northern Maine. Within the 904 towns with sufficient WT data, 756 towns had sufficient FIA plot density. In all, 701 towns met the sampling density threshold for both the WT and FIA data. We used only these 701 towns in all subsequent analyses. The average density of WTs in the final sample was 1.77 trees per km^2^ (*s* = 2.52) and 252 trees/town (*s* = 357). The average density of FIA plots in the final sample was 0.27 plots per km^2^ (*s* = 0.10) and 4.26 plots per town (*s* = 2.44). The average density of FIA trees was 0.93 trees per km^2^ (*s* = 0.41) or 156 trees per town (*s* = 86). Historical and modern tree data and town shapefiles are available at the Harvard Forest data archive (http://harvardforest.fas. harvard.edu/data/archive.html) as data set HF-210.

**Figure 3:**
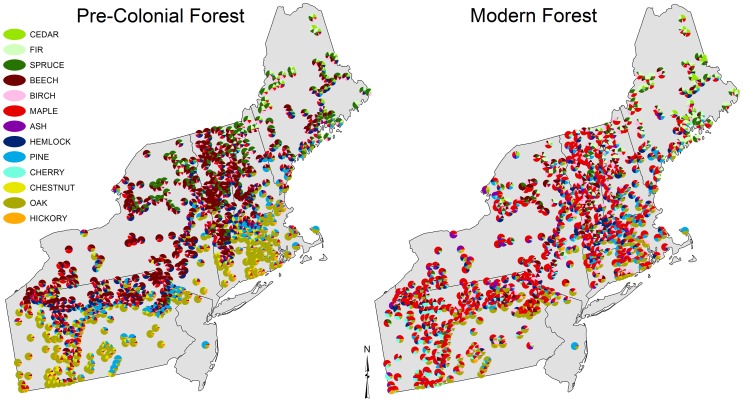
Relative composition of pre-colonial era Witness Trees and modern inventory trees in 701 colonial townships in the northeastern USA.

**Figure 4:**
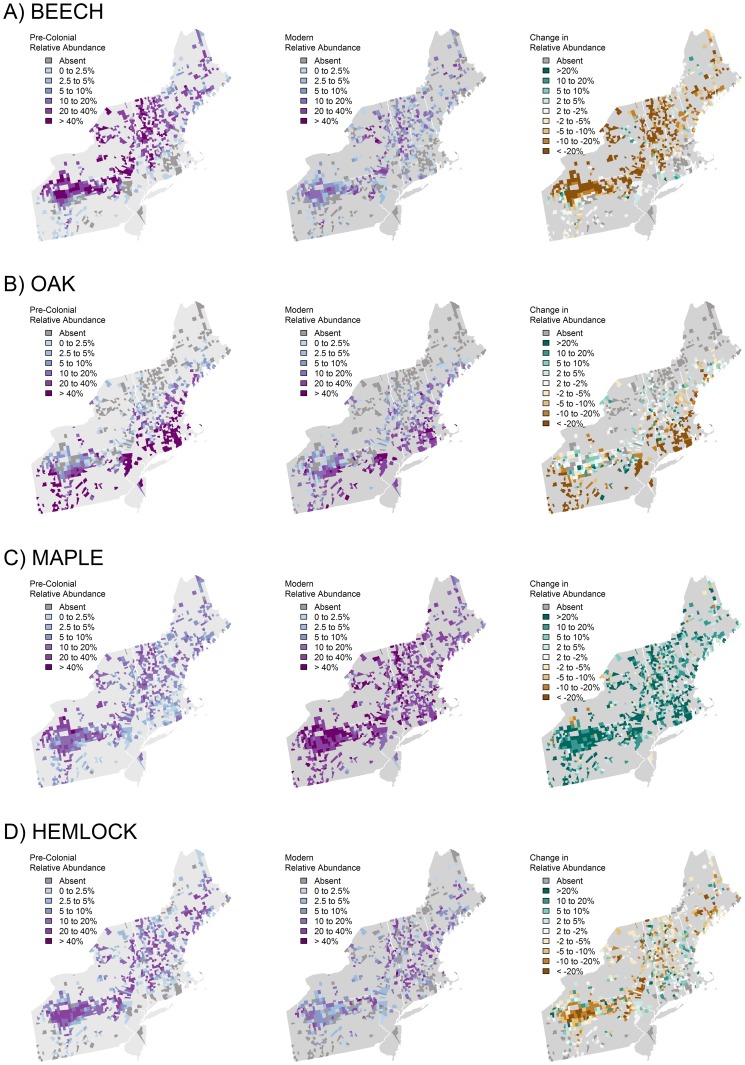
Town-scale relative abundance of forest taxa within 701 colonial townships. Pre-colonial (left); Modern taxa (center); change in abundance between the eras (right), A. Beech; B. Oak; C. Maple; D. Hemlock. Other taxa can be found in online supplementary materials.

While most taxa persist, the modern forest is compositionally distinct from the pre-colonial condition (Figure 3). Across the region, beech experienced the largest decline in relative abundance from the pre-colonial to modern era (Figure 4), dropping from an average of 22 percent to seven percent (Table 3). Major changes in beech were clustered in VT, western MA, and northern PA. Beech abundance was relatively stable in the Adirondack Mountains of NY. Oaks also underwent substantial declines in abundance, from 18 percent in the pre-colonial data to 11 percent in the modern data. Oak declines were most pronounced in central MA and southwestern PA. Hemlock declined from 11 to seven at a regional scale. Chestnut trees were extirpated from the modern forests, dropping from three to zero percent region-wide (Figure S1). The loss was most pronounced in the Appalachian Forest ecoregion where its pre-colonial abundance was ten percent. Due mostly to changes in the north, the abundance of spruce declined overall from 7.6 to 4.0 percent while fir increased from 2.0 to 4.5 percent. Maples experienced the highest absolute change in relative abundance, but unlike the previously mentioned taxa, average maple abundance increased throughout the region, from an average of 11 to 31 percent. Cherries also increased in relative abundance; climbing from <0.4 to 4.4 percent (Figure S1).

**Table 3:** Pre-colonial and modern relative abundance of taxa by ecoregion and throughout the entire study area.

	LAUREN (n = 222)			ADIRON (n = 242)			BROAD (n = 177)			APPAL (n = 60)			ALL (n = 701)		
	Pre	Mod	Δ	Pre	Mod	Δ	Pre	Mod	Δ	Pre	Mod	Δ	Pre	Mod	Δ
ASHES	2.0%	4.6%	2.7%^†††^	1.9%	3.7%	1.8%^†††^	2.9%	4.8%	1.9%^†††^	1.1%	1.3%	0.2%	2.1%	4.0%	1.9%^†††^
BASSWD	1.3%	0.7%	−0.6%^†††^	1.0%	0.3%	−0.7%^†††^	1.1%	0.5%	−0.6%^†††^	0.6%	1.0%	0.4%	1.1%	0.5%	−0.5%^†††^
BEECH	26.6%	6.6%	−20.0%^†††^	30.3%	9.9%	−20.4%^†††^	9.7%	2.3%	−7.4%^†††^	7.2%	5.0%	−2.3%^†^	22.0%	6.5%	−15.4%^†††^
BIRCHES	7.7%	8.3%	0.5%	10.1%	14.2%	4.0%^†††^	3.0%	7.9%	4.9%^†††^	2.5%	7.7%	5.1%^†††^	6.9%	10.2%	3.2%^†††^
BLKGUM	0.1%	0.2%	0.1%	0.0%	0.0%	0.0%	0.3%	0.5%	0.2%	1.0%	3.4%	2.4%^†††^	0.2%	0.5%	0.3%^†††^
CEDARS	2.3%	3.6%	1.3%^†^	1.0%	0.7%	−0.3%	0.0%	0.5%	0.5%^†††^	0.0%	0.0%	0.0%	1.1%	1.5%	0.4%^†^
CHERRIES	0.4%	5.3%	4.9%^†††^	0.2%	2.3%	2.1%^†††^	0.4%	4.6%	4.1%^†††^	0.4%	8.5%	8.1%^†††^	0.4%	4.4%	4.0%^†††^
CHESTNUT	2.6%	0.0%	−2.6%^†††^	0.8%	0.0%	−0.8%^†††^	5.5%	0.0%	−5.4%^†††^	9.5%	0.0%	−9.5%^†††^	3.3%	0.0%	−3.3%^†††^
CYPRES	0%	0.0%	0%	0.0%	0.0%	0.0%	0.1%	0.1%	0.0%	0.0%	0.0%	0.0%	0.0%	0.0%	0.0%
ELMS	0.8%	0.4%	−0.4%	0.8%	0.4%	−0.4%^†††^	1.1%	1.4%	0.3%	0.2%	0.2%	0.0%	0.8%	0.6%	−0.2%
FIRS	2.3%	6.3%	4.0%^†††^	3.7%	6.9%	3.2%^†††^	0.1%	0.4%	0.4%^†^	0.0%	0.0%	0.0%	2.0%	4.5%	2.5%^†††^
HEMLCK	14.8%	8.0%	−6.7%^†††^	12.8%	8.3%	−4.5%	5.4%	7.6%	2.2%^†^	5.4%	2.3%	−3.1%^†^	10.9%	7.5%	−3.4%^†††^
HICKORIES	0.9%	0.8%	−0.1%	0.3%	0.3%	0.0%	6.2%	3.1%	−3.0%^†††^	5.8%	2.0%	−3.7%^†††^	2.4%	1.3%	−1.1%^†††^
HORNBM	1.3%	1.3%	0.0%	1.0%	1.3%	0.3%	1.3%	0.7%	−0.5%^†^	0.6%	0.3%	−0.3%	1.1%	1.1%	0.0%
MAGNOL	0.2%	0.1%	−0.1%^†^	0.0%	0.0%	0.0%	0.2%	0.1%	0.0%	0.4%	0.8%	0.3%	0.1%	0.1%	0.0%
MAPLES	12.6%	30.9%	18.2%^†††^	12.2%	32.4%	20.1%^†††^	9.2%	29.2%	20.0%^†††^	8.6%	28.5%	19.9%^†††^	11.3%	30.8%	19.5%^†††^
OAKS	9.0%	8.4%	−0.6%	3.8%	4.3%	0.5%	40.5%	18.4%	−22.1%^†††^	37.2%	27.6%	−9.6%^††^	17.5%	11.1%	−6.5%
PINES	6.5%	5.3%	−1.2%	3.1%	6.3%	3.2%^†††^	9.8%	11.3%	1.5%	14.2%	3.4%	−10.8%^†††^	6.8%	7.0%	0.2%
POPLARS	0.6%	2.9%	2.3%^†††^	0.5%	1.6%	1.1%^†††^	0.9%	2.4%	1.6%^†††^	0.2%	0.7%	0.5%	0.6%	2.1%	1.5%^†††^
SPRUCES	6.5%	4.6%	−1.9%^†††^	15.4%	6.6%	−8.8%^†††^	0.7%	0.8%	0.2%	1.0%	1.0%	−0.1%	7.6%	4.0%	−3.6%^†††^
SYCMOR	0.2%	0.0%	−0.2%	0.0%	0.0%	0.0%	0.1%	0.0%	−0.1%^†††^	0.1%	0.0%	−0.1%	0.1%	0.0%	−0.1%^†††^
TAMRAC	0.1%	0.3%	0.2%	0.2%	0.2%	−0.1%	0.0%	0.1%	0.1%	0.0%	0.0%	0.0%	0.1%	0.2%	0.0%
TULIP	0.1%	0.1%	0.0%	0.0%	0.0%	0.0%	0.4%	1.0%	0.6%^††^	0.0%	0.0%	0.0%	0.2%	0.4%	0.2%^†††^
WALNUTS	0.3%	0.0%	−0.2%^†††^	0.2%	0.0%	−0.2%^†††^	0.2%	0.2%	0.0%	0.4%	0.1%	−0.4%^†††^	0.2%	0.1%	−0.2%^†^

P-values were derived from a paired Monte Carlo test that describes the probability of encountering differences in relative abundance that were at least as large as those observed from chance alone, given the distribution of data. ^†^ p<0.05; ^††^ p<0.01; ^†††^p<0.001.

**Figure 5:**
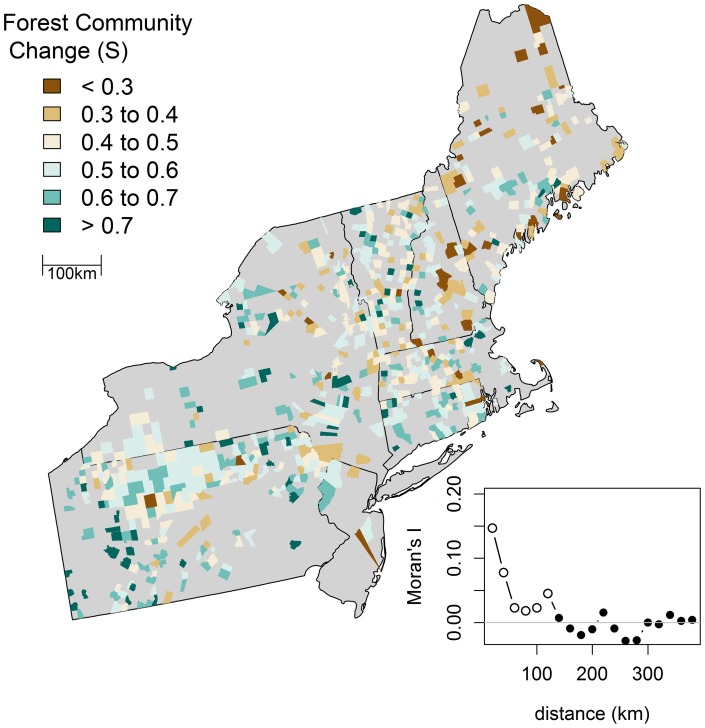
Town level compositional change (Sørensen's distance) between the pre-colonial and the modern forest taxa. Higher values indicate greater compositional change over time. Inset: Spatial autocorrelation in compositional change (Sørensen's distance) over time as shown in a Moran's I spatial correlogram with 20 km distance classes. Solid points indicate that spatial autocorrelation in that distance class is significantly different than zero (P<0.01).

The degree of overall compositional change from the pre-colonial to modern forests varied widely from town to town (Figure 5). Sørenson's values ranged from 0.13 to 0.94 (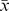 = 0.54, *s* = 0.131). The Central Appalachian ecoregion had the highest average compositional change (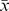 = 0.558, *s* = 0.12), followed by the Eastern Broadleaf Forest (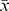 = 0.551, *s* = 0.15), then the Laurentian Mixed Forest (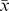 = 0.517, *s* = 0.13) and finally the Adirondack-New England Mixed Forest (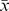 = 0.494, *s* = 0.12); however, all pair-wise comparisons between ecoregions were statistically insignificant. The Moran's spatial correlogram showed evidence of clustering in the degree of compositional change at short to moderate geographical distance (<125 km) – i.e., towns that incurred a high degree of compositional change tended to be surrounded by towns that also incurred a high degree of change, and vice versa (Figure 5). At distances >125 km there was no evidence of spatial patterning (i.e., autocorrelation).

**Figure 6:**
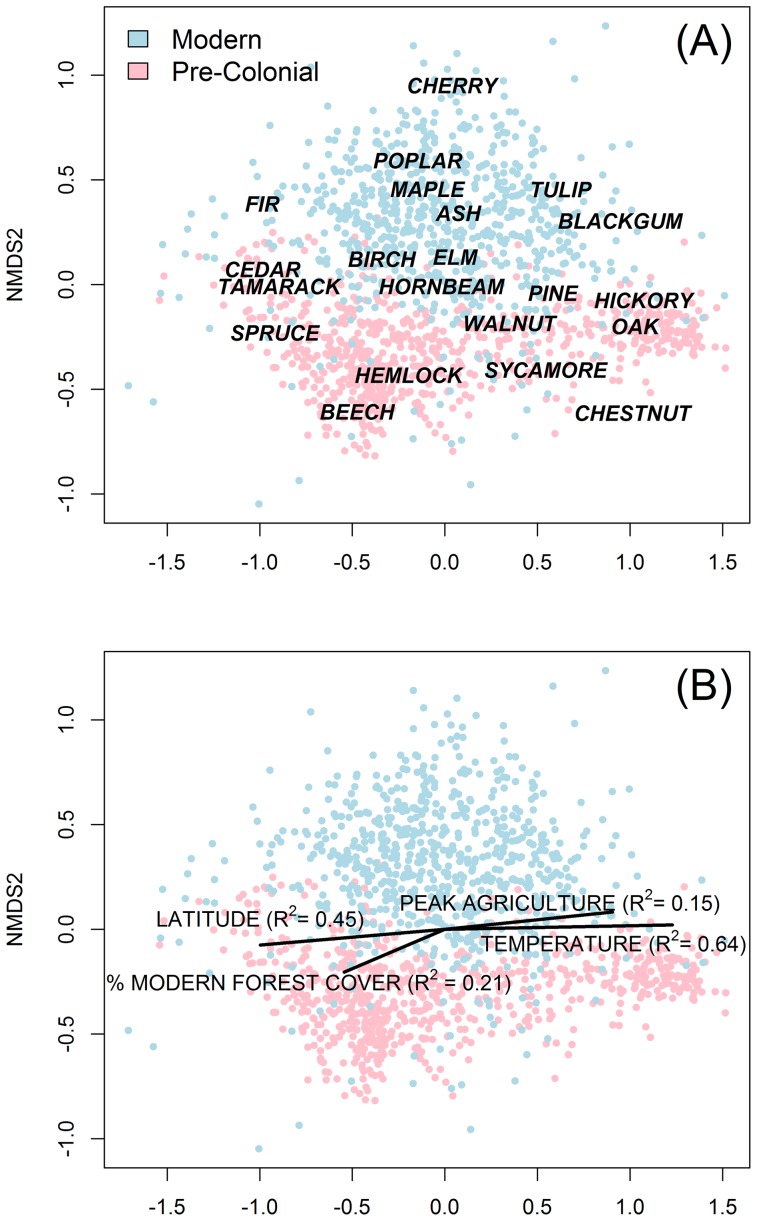
Non-metric multidimensional scaling ordination of pre-colonial and modern forest composition. (A) Points represent each town in each time period. Taxa names are position at the centroid of their distributions within the ordination. (b) Environmental parameters (Table 3) were overlaid onto the NMDS ordination diagram as fitted vectors. R-Square describe the correlation between ordination axes and environmental vectors only vectors with significant Pearson correlation (P<0.05.) were plotted.

In the two-dimensional NDMS ordination (Stress  = 9.56) compositional differences between the colonial and modern periods are apparent as directional shifts along Axis 2 (Figure 6). Ninety thee percent (653/701) of the town's pre-colonial data have Axis 2 scores <0. Conversely, 86 percent (604/701) of the town's modern data have Axis 2 scores >0. The overlay of taxa centroids onto the ordination further demonstrated the differences in composition along Axis 2. For example, cherry had the highest Axis 2 values and chestnut had the lowest (Figure 6a). Axis 1of the ordination captured compositional gradients related to climate Average temperature (i.e., growing degree days) and latitude had the highest correlation with Axis 1 (Figure 6b). Climatic influence on forest composition was also evident in the placement of taxa centroids along Axis 1. For example, the centroid for oak – a taxa more common in the southern part of the study area – scored quite high on Axis 1, while spruce and fir – both northern taxa – scored quite low. Chestnut was placed high on Axis 1, corresponding to its greater abundance in the southern townships in our study area, while also scoring low Axis 2 due to its absence from modern forests.

**Figure 7:**
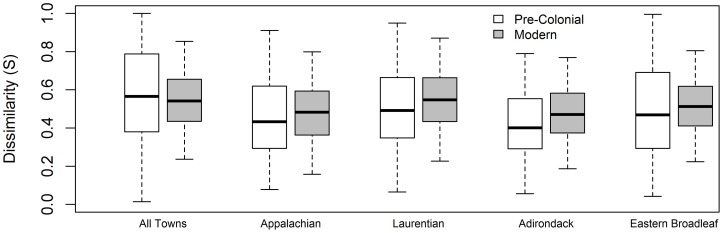
Changes in landscape β diversity between the pre-colonial and the modern forest taxa as shown though the distribution of town-to-town compositional dissimilarities (Sørensen's distance). Higher values correspond to higher β diversity. The box represents the inner quartile range of inter-town compositional dissimilarity. The horizontal line indicates the median. The whiskers extend to 1.5 times the inner quartile range.

At the full regional scale, average town-to-town dissimilarity was higher in the pre-colonial forests (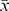 = 0.58, *s* = 0.24) than in the modern forests (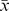 = 0.55, *s* = 0.16; Figure 7). In contrast, in each of the ecoregions average town-to-town dissimilarity was higher in the modern forests than in the pre-colonial. Differences were highest in the Laurentian Mixed Forest ecoregion (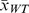 = 0.51 *v.* 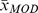 = 0.56), followed by Adirondack-New England Mixed Forest ecoregion (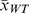 = 0.43 *v.* 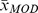 = 0.48), then the Eastern Broadleaf Forest ecoregion (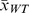 = 0.50 *v.* 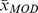 = 0.52) and finally the Central Appalachian Ecoregion (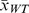 = 0.46 *v.* 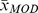 = 0.49). All comparisons between average pre-colonial and average modern data were significantly different (P<0.001), though, this reflects the high degrees of freedom associated with all pair-wise town-to-town dissimilarities and does not necessarily imply that the differences were biologically significant.

**Figure 8:**
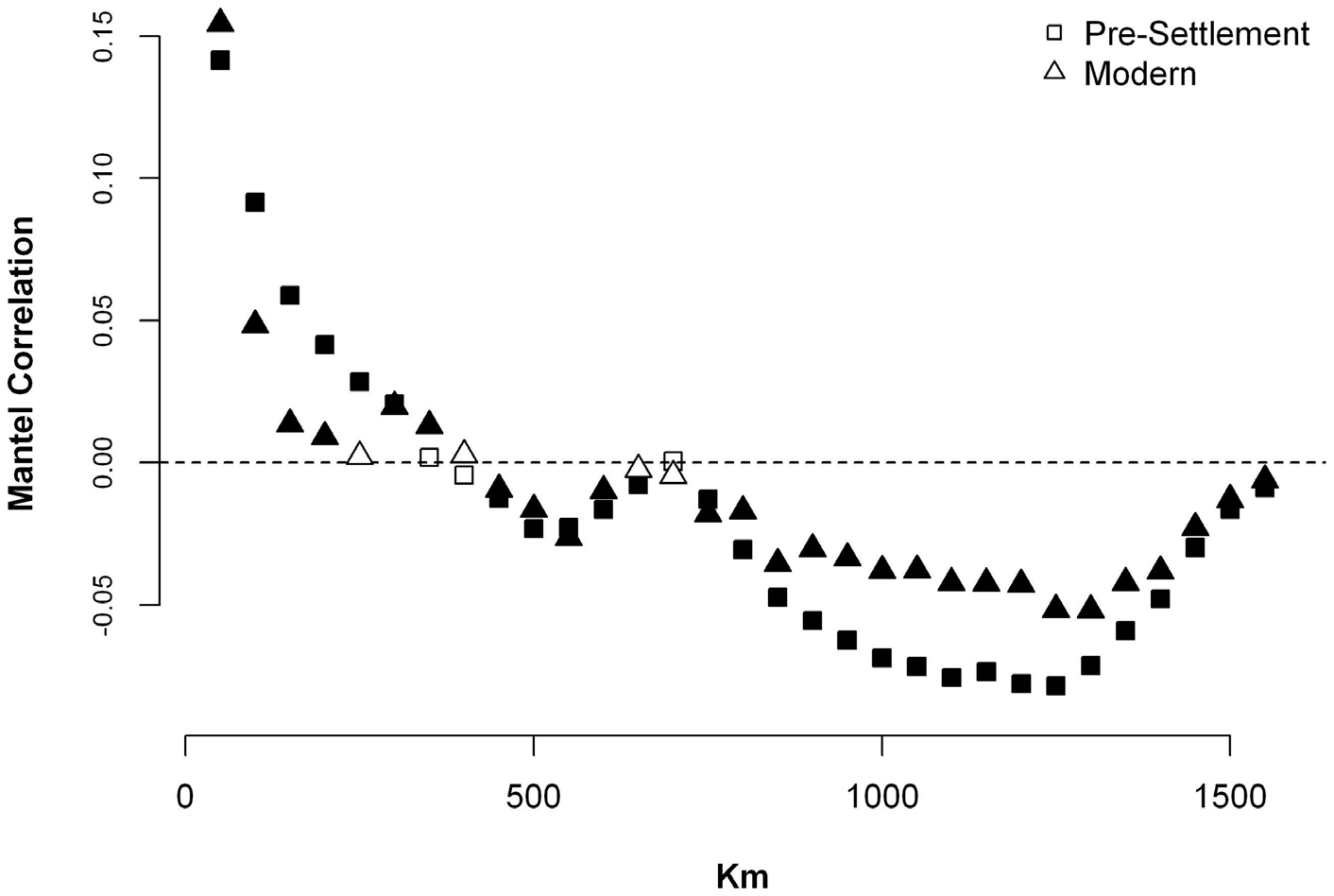
Mantel correlogram showing the spatial correlation for both the pre-colonial (squares) and modern (triangles) forest composition. Filled symbols indicate that the correlation is significantly different than zero using a Bonferroni adjusted α of 0.05. Significant positive correlations indicate towns that are separated by the geographic distance indicated on the x-axis are compositionally more similar than would be expected by chance, while significant negative correlations indicate that towns are more dissimilar than would be expected by chance.

The Mantel correlogram showed that pre-colonial forest composition had significant positive spatial correlation from 1 to 400 km and significant negative spatial correlation from 400 to 1500 km (with the exception of sites roughly 700 km apart)–i.e., towns <400 km apart were compositionally more similar than would be expected by chance and towns that were >400 km apart were more dissimilar than would be expected by chance (Figure 8). The modern forest composition followed a roughly similar pattern, except that the strength of compositional clustering and dispersion were not as strong. Mantel correlations between community composition and environmental data varied widely (Table 4). Annual temperature (i.e., growing degree days) had the highest correlations with forest composition in both time periods, but the correlation with the pre-colonial composition was much higher (0.60) than with the modern composition (0.34). The *r*_M_ values for elevation and percent sand were much weaker (<0.2) in both eras. There was no significant correlation between composition and annual precipitation in either time period.

**Table 4:** Mantel *r_M_* correlations between climatic, topographic, and edaphic variable (Table 2).

	Pre-Colonial	Modern
Temperature GDD	0.60^†††^	0.34^†††^
Precipitation	NS	NS
Elevation	0.13^†††^	0.14^†††^
Sand	0.07^†††^	0.09^†††^

P-values are derived from a Monte Carlo test that describes the empirical probability that the correlations are significantly different than zero. † p<0.05; †† p<0.01; †††p<0.001; NS  =  Not Significant.

**Figure 9:**
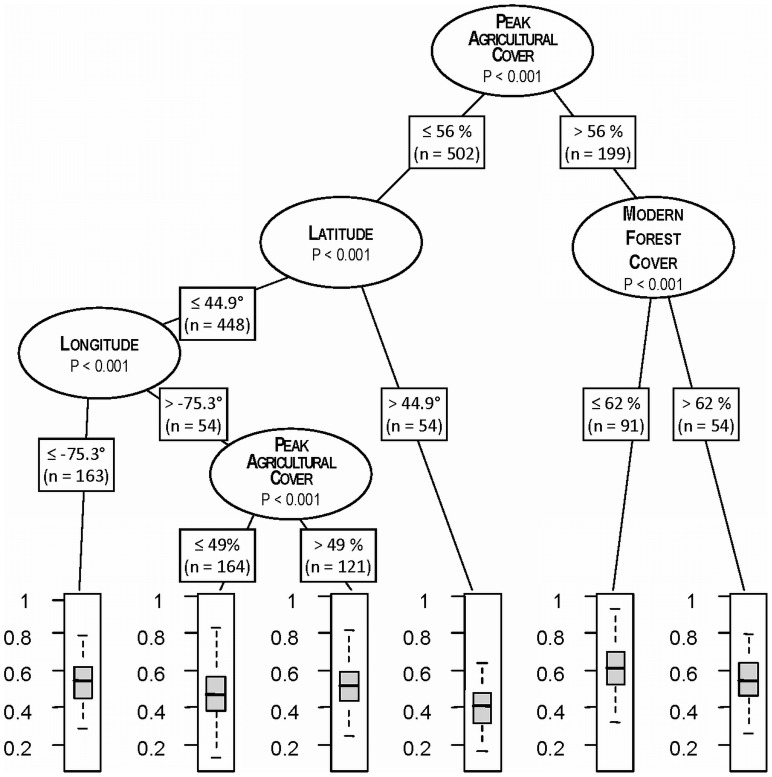
Conditional inference tree showing a hierarchy of relationships between the potential predictor variables (Table 3) and the degree of compositional change (Sørensen's distance) between the pre-colonial and the modern forest taxa (Figure 4). Partitions in the dendrogram represent the lowest statistically significant P-value that is obtainable across all levels of all predictor variables.

The RTA identified five significant partitions using four different predictor variables (Figure 9). The first partition was based on whether the maximum percent of a town historically in agriculture was greater or less than 56 percent. The group with greater extent of agriculture experienced, on average, significantly higher levels of compositional change. The model further partitioned the high-agriculture group based on whether modern forest cover was greater or less than 62 percent; the less forested of these groups had the highest level of compositional change across all terminal nodes in the tree. The towns with lower levels of agriculture was next partitioned based on latitude, with towns north of 44.9° experiencing the lowest level of compositional change across all nodes in the tree. The towns with a lower level of agriculture but south of 44.9° were again partitioned based on whether the town longitude was east or west of −75.3. The more westerly group was split again based on the maximum percent of a town historically in agriculture. Towns where the maximum proportion of land in agriculture was greater than 49 percent had higher compositional change.

## Discussion

Our analyses document a remarkable paradox about the eastern forest after 400 years of land use: it is at once largely unchanged and completely transformed. It is unchanged insomuch as all the major arboreal taxa remain. With few exceptions, the same taxa that made up the forest in the pre-colonial period comprise the forest today, despite ample opportunities for species invasion and loss. In this sense, the regional ecosystem has been quite resilient and the recovery of the eastern forests has been quite real in extent and composition. Yet, at the same time, the forest has been radically transformed. The relative abundance and distribution of most taxa have shifted dramatically; the relationship between forest composition and the environment has been weakened; and variable patterns of land use have imposed a mosaic of impacts whose legacies are evident centuries later. In the discussion that follows we address the nature of these changes in the context of our research questions, starting with the broad-scale compositional changes and working toward specific trends from individual taxa.

### The scale, pattern, and correlates of compositional change

Forest composition changed throughout the region – from Maine to Pennsylvania – and no single ecoregion incurred a significantly higher or lower magnitude of change. The degree and ubiquity of compositional change is clear in the ordination (Figure 6), which showed remarkably little overlap in the distribution of modern and pre-colonial towns. It is important to note, however, that this ‘ubiquitous change’ manifested quite variably at sub-regional scales and that the actual changes – i.e., the rise and fall of individual taxa – occurred non-uniformly. Specific compositional shifts were linked to the specific flora and land-use history of individual towns or small clusters of towns. Indeed, we saw strong local-scale (<125-km) clustering in the magnitude of compositional change but almost no evidence of regional-scale gradients in that change (Figure 2).

Why do we see clusters of two to five towns with similar levels of change? The regression tree analysis suggested that local-scale clustering of compositional change may be explained by a hierarchy of land use history and climatic factors (Figure 9). The modern forests arose following land uses of varying types and intensities that had direct impacts on composition–such as forest harvesting , land clearance and agriculture – and indirect impact, through interactions among land use, climate and the biophysical setting. These different effects are apparent in the form of the regression tree. The first partition – and therefore the variable with the greatest explanatory power – is based on the maximum level of agricultural clearing between 1850 and 1997. Towns with less than 56 percent of their area cleared for agriculture experienced significantly less compositional change. The emergence of agricultural land clearing as the most important variable provides strong evidence of the effects of land use on long-term compositional change. The enduring impact of land use on composition has also been documented in some smaller scale witness tree studies [29], [37], [49] and some detailed field-based studies that document the strong legacy of agriculture versus managed woodlot on modern vegetation [50], [51].

Among the towns with higher agricultural clearing, the level of modern forest cover is the next best predictor of change. Towns that have largely reforested (>62%) exhibit less compositional change than those with high levels agriculture of other developed land covers. Among the towns with lower agriculture cover, the next split in the regression tree is based on latitude with the northerly sites – those in northern Maine – having the lowest level of change across the entire region. Towns with lower historical agriculture but south of Maine were next partitioned based on latitude; towns west of Scranton, PA (75.3°) are less changed, perhaps because these towns were colonized later and, therefore, have a shorter history of land use than towns to the east.

### An overall homogenization and de-coupling of composition and environment

The modern forest composition is less coupled to its local environment than was the pre-colonial forest, which has coincided with a broad-scale compositional homogenization (sensu [52]). Among the suite of environmental variables we examined, temperature had, by far, the strongest association with forest composition in both time periods; however, the strength of that association was dramatically lower in the modern era (*r_M_* = 0.60 versus *r_M_* = 0.34; Table 4). This has manifested as a compositional “smoothing” across the region. This also affected the inter-town compositional dissimilarity within each time period, which we used as a straightforward comparison of β-diversity (Figure 7). At the full regional scale, mean dissimilarity was marginally higher in the pre-colonial forests – i.e., the community composition between any two towns, on average, is slightly more similar in the modern era than it was in the colonial period. In contrast, at the ecoregional scale, towns were compositionally more similar to each other in the pre-colonial era than in the modern era. Perhaps more telling than the averages, though, were the differences in variation. At the regional scale, the variance was more than twice as high in the pre-colonial era, where many town-pairs had Sørensen values close or equal to one (indicating little or no overlap in taxa) and many towns had values close to zero (indicating similar taxa in similar abundances). In contrast, the distribution of modern inter-town dissimilarities was comparatively concentrated around the mean.

The regional biotic homogenization is visually apparent in mapped community composition (Figure 3). In the pre-colonial maps, the towns in the south are dominated by oak and hickory and have little in common with the northern towns dominated by spruce and fir. In contrast, most modern towns have a significant component of maple regardless of their location. We confirmed this empirically and showed that, indeed, pre-colonial forests were compositionally more similar at short distances (i.e., positive spatial autocorrelation in the Mantel correlogram (Figure 5)) and more dissimilar at longer distance (i.e., negative spatial autocorrelation).

Smaller scale studies have arrived at opposing conclusions with regard to homogenization of the modern forest. For example, in western NY, the replacement of a late successional species (beech) with several early successional species resulted in increased compositional heterogeneity [27]. In contrast, Foster et al [4] observed a pronounced homogenization and the loss of affinity between taxa abundances and the regional climate gradient in central MA. Such divergent conclusions regarding the impact of historical land use underscore the importance of considering the impacts of a regional-scale disturbance regime at the regional scale. From our analysis, it seems clear that the regional ecosystem has not reestablished the mosaic of forest-types and strong climate-driven compositional gradients.

### A broad ecological shift from late to early successional taxa

Our data offer snapshot perspectives on the pre-colonial and modern forest composition. From these we can make inferences about the succession and disturbance processes that shaped the forests. For example, that late successional species such as beech and hemlock were common in the pre-colonial data is a testament to the relative stability of these forests and their disturbance regimes for the millennia preceding colonization. Beech and hemlock are archetypically late successional species; they are shade tolerant, slow growing, long-lived, and slow to re-colonize a site after disturbance. Based on their pre-colonial abundance, it is clear that the disturbance regime was long dominated by small canopy gaps.

The abundance of late successional species is significantly lower on the modern landscape, which offers evidence of the legacy of past land use and to changes in the modern disturbance regime. Of all the taxa we examined, beech experienced the most dramatic reduction in relative abundance, declining in 91 percent of the towns in which its pre-colonial abundance was greater than five percent. We found just one pocket of beech stability (or, at least, low change) located in the Adirondack Mountains of New York, which is the region within our study area that has the most old-growth and primary forest and has had the least exposure to human induced disturbances. This suggests that while climate and disease (particularly beech bark disease) may be contributing factors, the primary cause of beech reduction locally and regionally is the disruption of the forest by deforestation, logging and fire.

The overall reduction in hemlock is significant but less stark and more multifaceted than that for beech. In the past 10,000 years, several dramatic declines in hemlock are evident in pollen data , always coinciding with periods of drought and aridity [53]–[55]. Therefore, hemlock would be expected to be under stress during the greater aridity that coincided with the early colonial period and consequently less able to recover from a regime of intense land use and selective harvesting for its tannins throughout the colonial period [2]. Indeed, an initial decline by hemlock in many pollen diagrams just before European settlement is followed by a more rapid decline with intensive land use [56]. In the modern era, hemlock is being decimated by the invasive hemlock wooly adelgid (HWA; *Adelges tsugae*). We saw the largest reductions of hemlock in the southern portion of our study area, where it frequently declined to zero, which is consistent with the northward spread of HWA from Richmond, Virginia since 1952 [57]. At present, cold winter temperatures appear to be limiting HWA's extension into northern New England [58].

On the other end of the successional spectrum are species such as red maple (*Acer rubrum*), black cherry (*Prunus serotina*), and aspens (*Populus spp*). Within our analysis maple had, by far, the largest absolute change in relative abundance – a nearly 20 percent rise in average regional abundance. Because the FIA data have species-level resolution, we know that the maple species that dominates the modern landscape is overwhelmingly red maple, a pioneer species with moderate to great shade tolerance and light, wind-dispersed seeds that readily invades open fields after farm abandonment as well as occupy forest gaps following disturbance. It sprouts readily following damage and has great edaphic amplitude ranging from saturated wetland soils to drier uplands. The ecological versatility of red maple allows it to occupy a broad range of edaphic and climatic conditions throughout our study area [59]–[61]. And, indeed, maple increased in 87 percent of the towns we examined, spanning all the environmental gradients within the region, as demonstrated by its central location within the cloud of modern data in the ordination (Figure 6).

Similarly, cherry is positioned squarely in the modern data cloud within the ordination; indeed, it has the highest position along axis two of all taxa (Figure 6). Cherry was absent in most of the pre-settlement surveys, but was present at low abundance throughout much of the modern forests. Thanks again to the resolution of the FIA data, we know that references to cherry in the modern data are mostly (>75%) black cherry. The prevalence of this relatively short-lived, pioneer taxon reflects the greater extent and intensity of forest disturbance in the modern era. It shows that disturbance, e.g., from harvesting and meteorological processes, is ongoing throughout the region and the macro shift to reforestation 150 years ago does not imply that succession has progressed uninterrupted.

In the absence of anthropogenic influences we would expect succession to increase the similarity to the pre-colonial forests. By examining our two snapshots of forest composition coupled with our understanding of past and ongoing disturbance processes, we can infer that this has not yet occurred because: (1) despite more the 150 years of reforestation, these ecosystems are still in the early stages of recovery, and (2) through that 150-year period there has been ongoing harvests and other land uses as well as exogenous disruptions, including pests (e.g., HWA), pathogens (e.g., chestnut blight), climate change, and atmospheric deposition.

### Dynamics of other significant taxa

Chestnut suffered a near complete extirpation as a tree-sized individual from the region due to the introduced fugal blight, *Endothia parasitica* in the early 1900s. Much has been written about the loss of chestnut and its replacement by oak and maple [28], [62]–[64]. Conventional wisdom holds that chestnut was among the dominant overstory species throughout eastern forests [65]–[67]. However, the average relative abundance of chestnut in the pre-colonial data was only 3.3-percent. Its highest abundance was in the Appalachian ecoregion, and even there it was less than ten percent and just one-quarter that of oak. Indeed, and in contrast to many other southern and northern taxa, no town in our analysis exceeded 25-percent pre-colonial chestnut abundance. These data suggest that the historical dominance of chestnut is currently often overstated, at least in the Northeast. Nevertheless, even with our comparatively modest estimate of pre-colonial abundance, the loss of chestnut initiated a significant change in forest composition.

Like chestnuts, oaks are mid- to late-successional, hard-mast producing canopy tree. There has been growing concern among researchers and managers that oaks are losing their dominance on the landscape [68], [69]. And, indeed, oaks declined in 75 percent of the towns in which its abundance was greater than five percent. Across the region, the relative abundance of oaks declined by almost seven percent. Declines were largest in the Eastern Broadleaf ecoregion where relative abundance fell from 40 to 18 percent. It is worth noting, however, that there were a few small pockets where the relative abundance of oak increased (e.g., in eastern PA) and there was little change along much of the northern extent of it pre-colonial range. McEwan et al. [69] recently dissected the myriad potential causes for the decline in oak; they conclude that a “multiple interacting ecosystem drivers hypothesis” is essential to understand long-term oak dynamics. Important factors include a climatic shift toward increased moisture availability, a shift away from Native American burning and a shift first toward low human populations and then to European colonization, plus changes in populations of acorn consuming fauna, including white tailed deer (*Odocoileus virginianus*).

The regional stability in average pine abundance belies the fact that pine was among the most dynamic taxa we examined (Figure S1). At the regional scale, the relative abundance of pine did not change significantly. However, in the Central Appalachian ecoregion, pines declined by 11 percent, a reduction that was offset by modest increases in pine in the other ecoregions. The relative abundance of pines increased by more than ten percent in 100 towns and decreased by more than ten percent in 99 towns. This high amplitude of change in pine abundance likely speaks to two countervailing characteristics of pine in the region: On one hand, pine is a pioneer taxon that establishes on disturbed sites including post-fire landscapes and abandoned agricultural fields, grows fast, and lives for a long time. On the other hand, pine is vulnerable to natural disturbance (particularly wind) and has always been among the most sought after timber species in the region [6], [70].

### Limitations of data sources

It is important to acknowledge the limitations of the datasets we employed and discuss how we dealt with them: (1) The WT data were collected over a period of more than 150 years. While the surveys were conducted at a similar timeframe relative to colonization, they still potentially represent different levels of Native American and European influence and different survey methods. We minimized our exposure to this by excluding towns with explicit evidence of colonial influence (e.g., those with apple trees) and by screening out the trees sampled using surveys types known to produce a bias (e.g., road surveys, *sensu* [71]). (2) The witness trees were not collected as a random sample. Instead we assembled all witness tree records known to exist with the nine-state region. While we know of no particular bias in the towns with existing data, we also cannot say emphatically that our collection of towns is a statistically robust sample. (3) The sample of WT and modern trees per town is sparse, which is why we put the data through an exhaustive analysis whereby we only retained towns where the WT and FIA data adequately captured the compositional diversity. (4) The FIA protocol measures in clusters whereas the WTs are dispersed throughout the town, which introduce potential for error and bias. We hedged against this by only including towns with at least two FIA plots and then putting the FIA plots through the rarefaction analysis. Having noted these issues, we still believe that historical survey records offer a singular resource for understanding widespread patterns of forest composition at the time of European colonization. This perspective has been borne out through several studies that have shown that these data provide an accurate account of landscape scale vegetation patterns (e.g. [2], [21]). In addition, consistency between WT data and contemporary pollen studies [56], [72] further confirm the utility of this resource.

## Conclusion

By comparing the modern forest condition to regional database of WT, we have learned much about the changes regional forest composition. While most native taxa remain, the community composition has shifted dramatically. While significant compositional changes were ubiquitous throughout the region, the specific attributes of change varied at local scales. One important pattern throughout was the reduction of late successional species in favor of early successional species. Additionally, the modern forest is more homogeneous and less coupled to local climatic controls.

Among individual taxa, we found that late successional trees such as beech, hemlock, and spruce, were once predominant across the region but are now much less so, save for in some small refugia within isolated mountain regions with little history of land use. Oaks also declined throughout most, but not all, of its range. The 400-year history of land use benefited maple the most and it is now a dominant taxon throughout most of the region. Short-lived, early seral species are also much more common in the modern landscape, which indicates that land clearing disturbance is ongoing and widespread and the abandonment of agriculture 150 years ago did not mark a return to the natural disturbance regime of small gap openings.

The northeast is once again a predominantly forested landscape, but today's forest is not a facsimile of its predecessor. We find this to be at once disheartening and encouraging. On the one hand, the modern expense of forest is diminished in so many of the components and processes that once characterized the regional ecosystem; on the other, given the extent and magnitude of land use it is remarkable that native species predominate and the forests looks in many ways as it has for millennia.

## Supporting Information

Figure S1 **Maps of relative abundance and change for all taxa.**
(ZIP)
