# Supplementary figures and images for: Four Centuries of Change in Northeastern United States Forests
Source: PLoS One. 2013 Sep 4;8(9):e72540. doi: 10.1371/journal.pone.0072540 (PMC3762820; doi:10.1371/journal.pone.0072540)

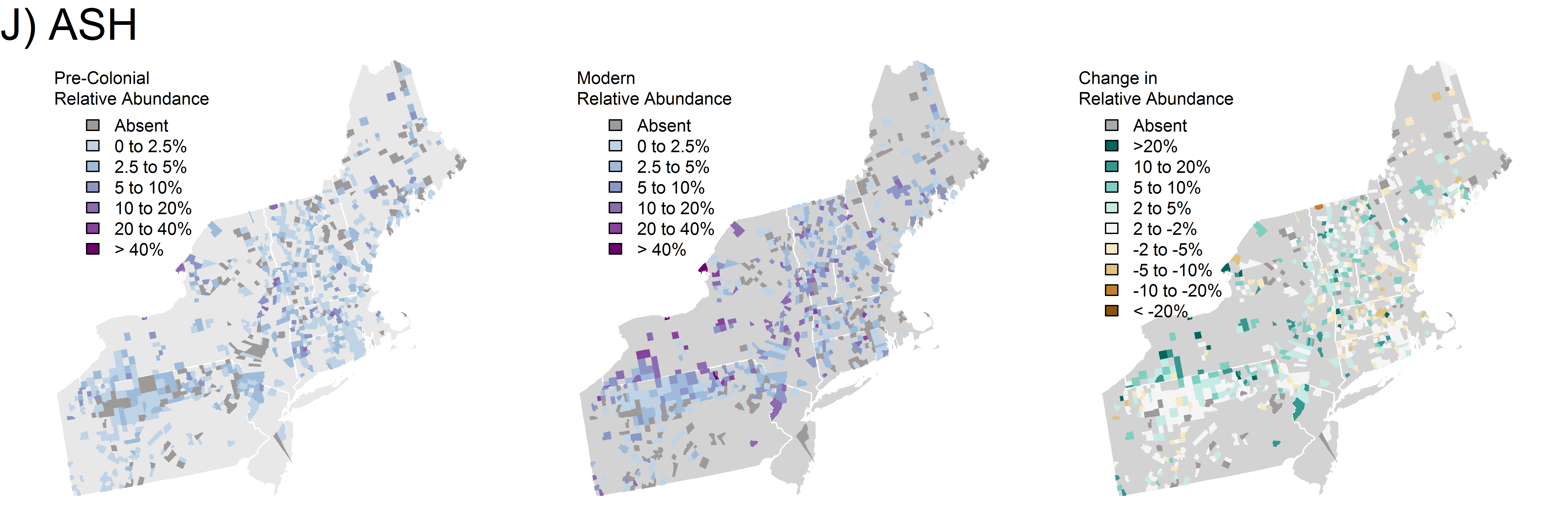

Supplement: Figure S1 — Maps of relative abundance and change for all taxa. (ZIP) [file pone.0072540.s001.zip › taxa_change_figs/ASHS.tif]

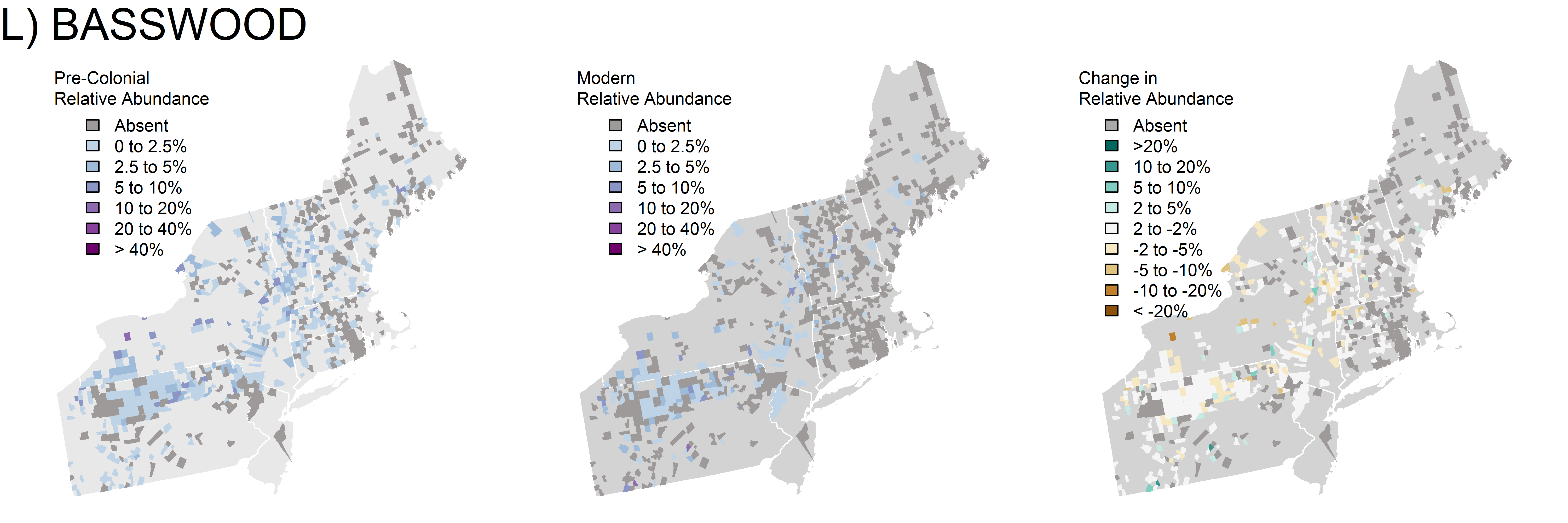

Supplement: Figure S1 — Maps of relative abundance and change for all taxa. (ZIP) [file pone.0072540.s001.zip › taxa_change_figs/BASSWD.tif]

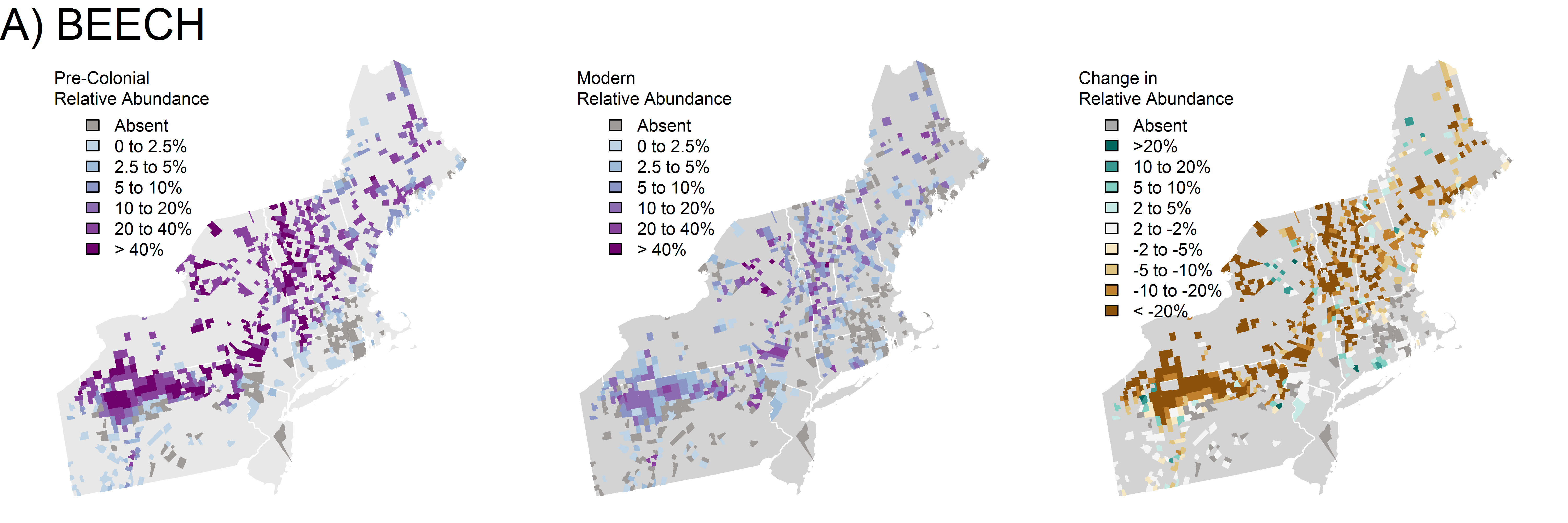

Supplement: Figure S1 — Maps of relative abundance and change for all taxa. (ZIP) [file pone.0072540.s001.zip › taxa_change_figs/BEECH.tif]

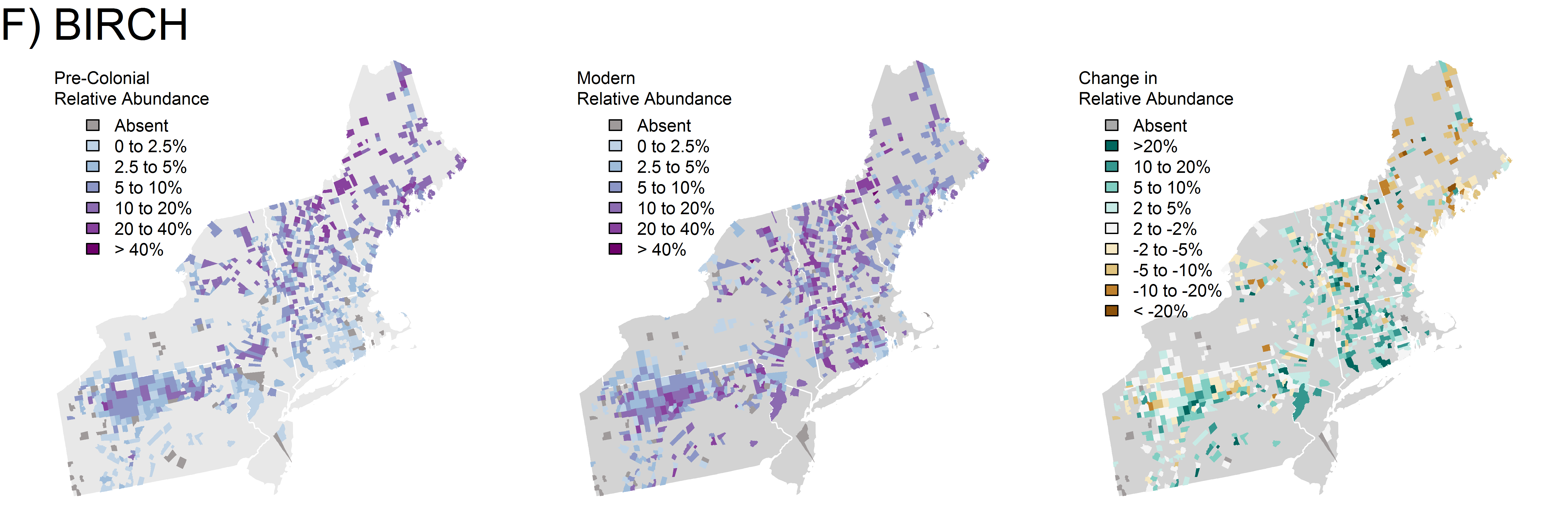

Supplement: Figure S1 — Maps of relative abundance and change for all taxa. (ZIP) [file pone.0072540.s001.zip › taxa_change_figs/BIRCHS.tif]

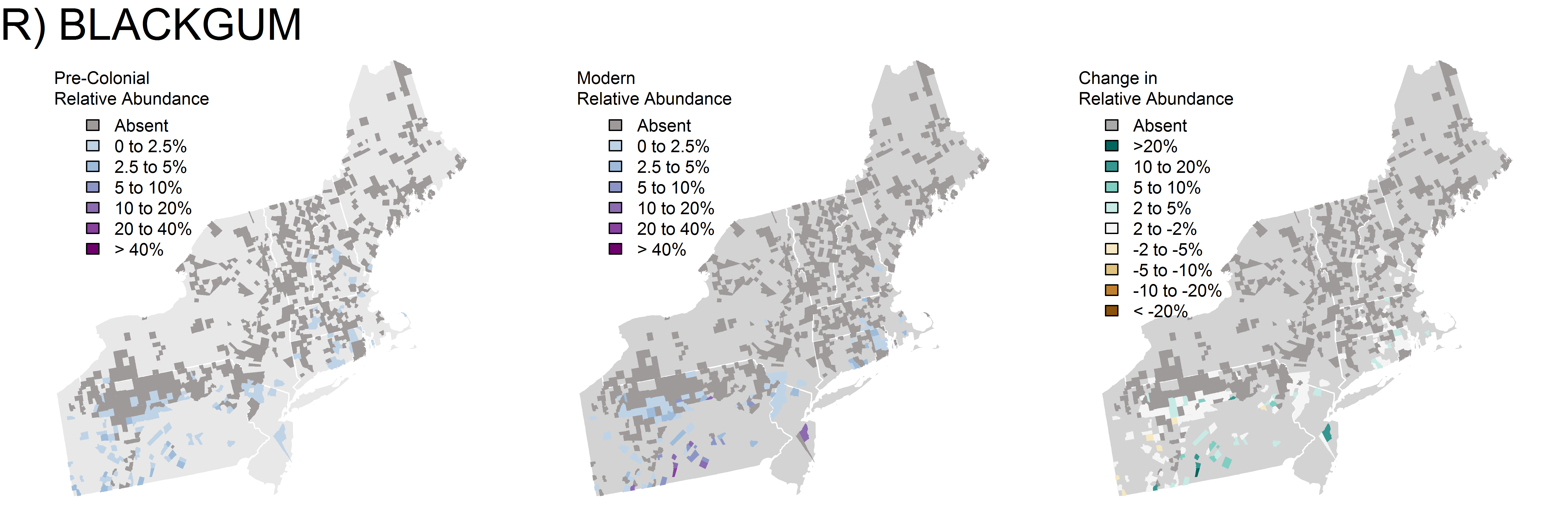

Supplement: Figure S1 — Maps of relative abundance and change for all taxa. (ZIP) [file pone.0072540.s001.zip › taxa_change_figs/BLKGUM.tif]

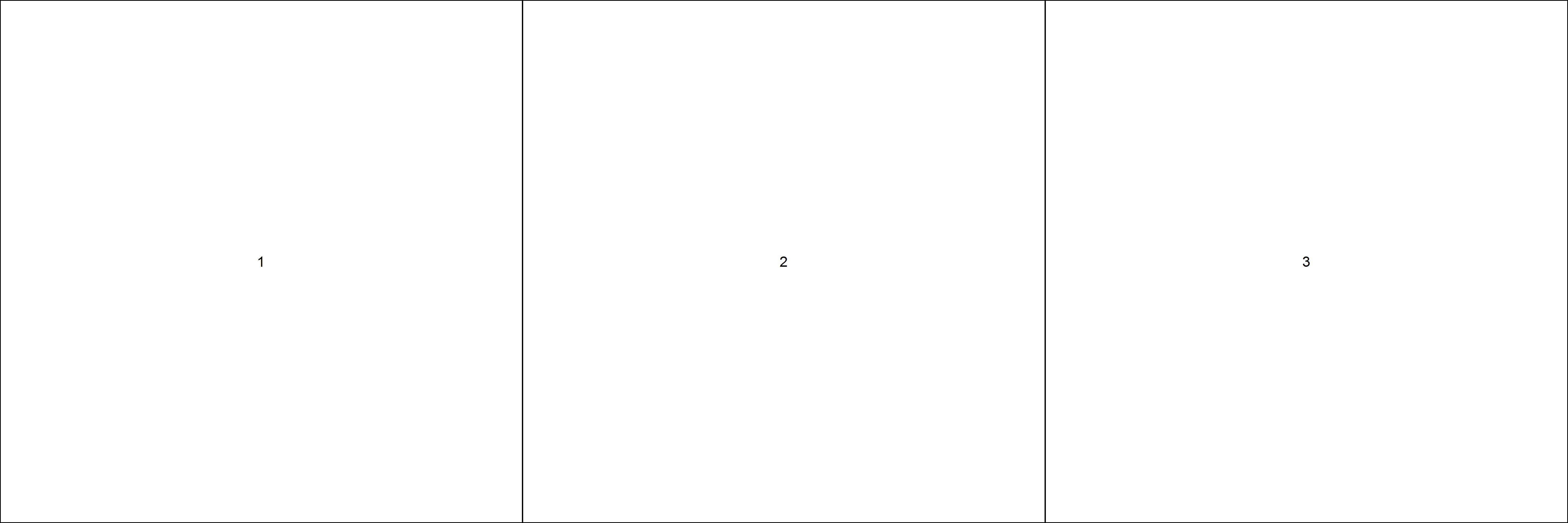

Supplement: Figure S1 — Maps of relative abundance and change for all taxa. (ZIP) [file pone.0072540.s001.zip › taxa_change_figs/CEDARS.tif]

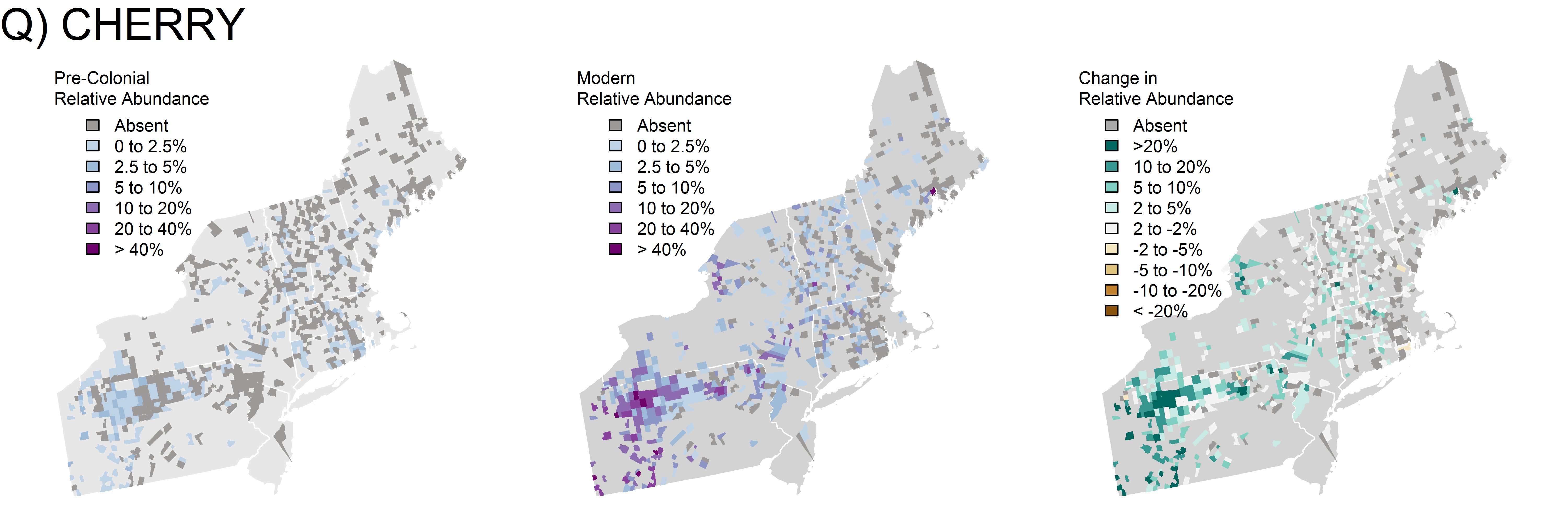

Supplement: Figure S1 — Maps of relative abundance and change for all taxa. (ZIP) [file pone.0072540.s001.zip › taxa_change_figs/CHERRY.tif]

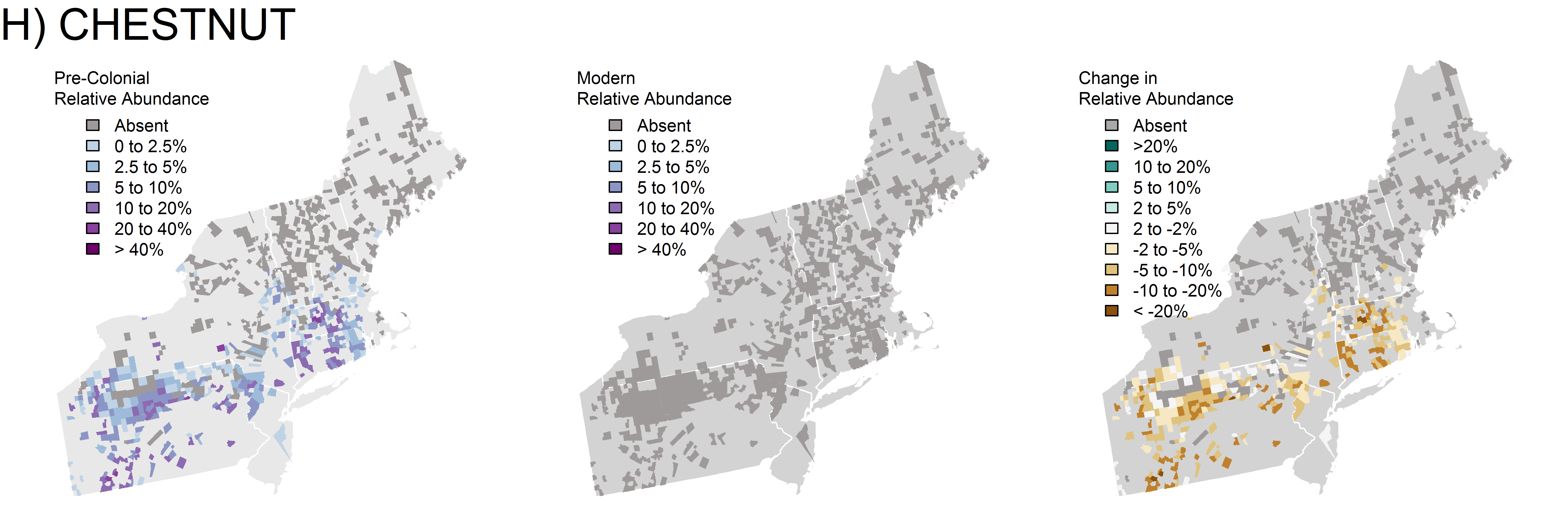

Supplement: Figure S1 — Maps of relative abundance and change for all taxa. (ZIP) [file pone.0072540.s001.zip › taxa_change_figs/CHSNUT.tif]

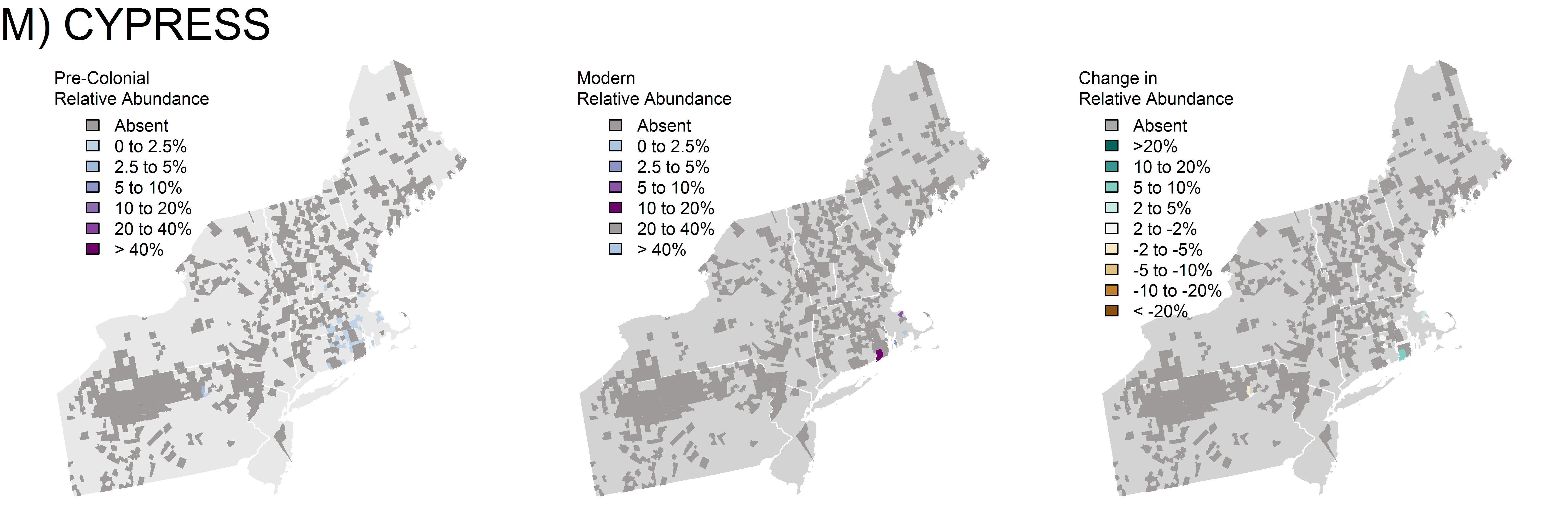

Supplement: Figure S1 — Maps of relative abundance and change for all taxa. (ZIP) [file pone.0072540.s001.zip › taxa_change_figs/CYPRES.tif]

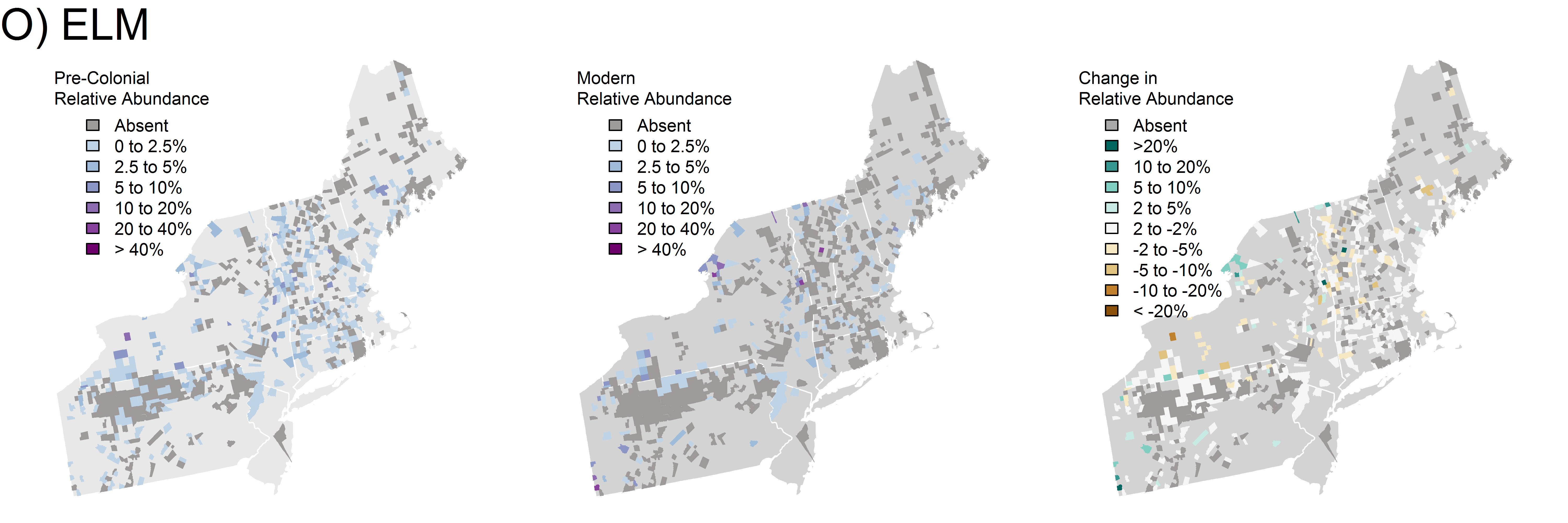

Supplement: Figure S1 — Maps of relative abundance and change for all taxa. (ZIP) [file pone.0072540.s001.zip › taxa_change_figs/ELMS.tif]

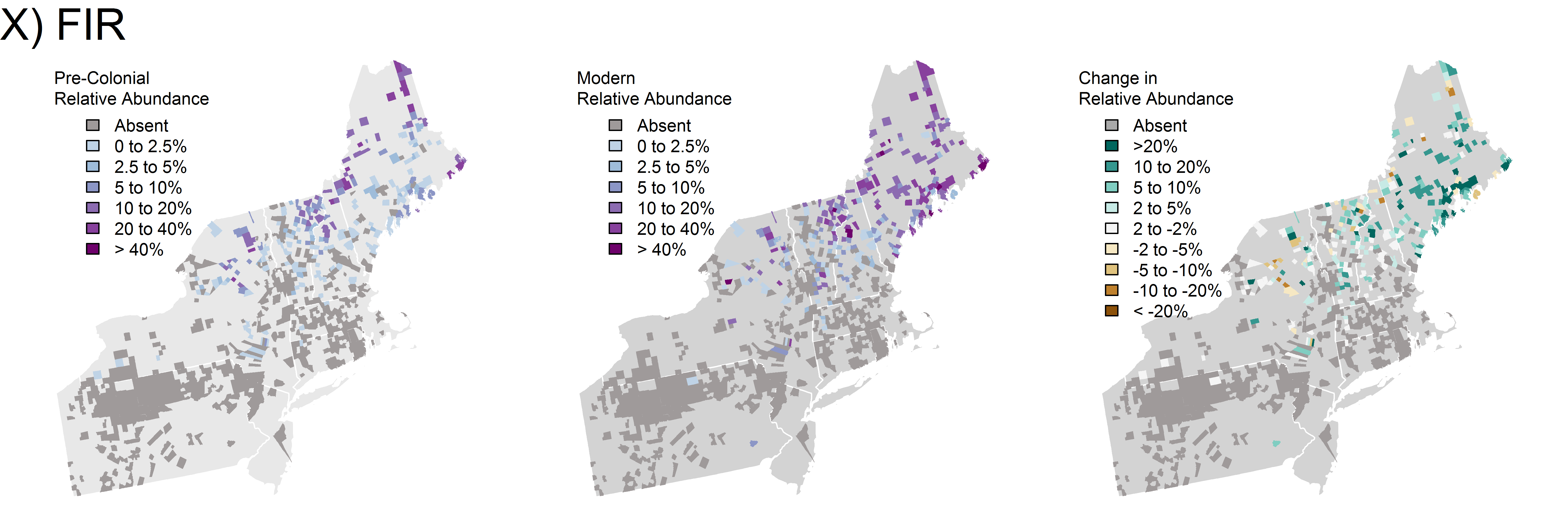

Supplement: Figure S1 — Maps of relative abundance and change for all taxa. (ZIP) [file pone.0072540.s001.zip › taxa_change_figs/FIRS.tif]

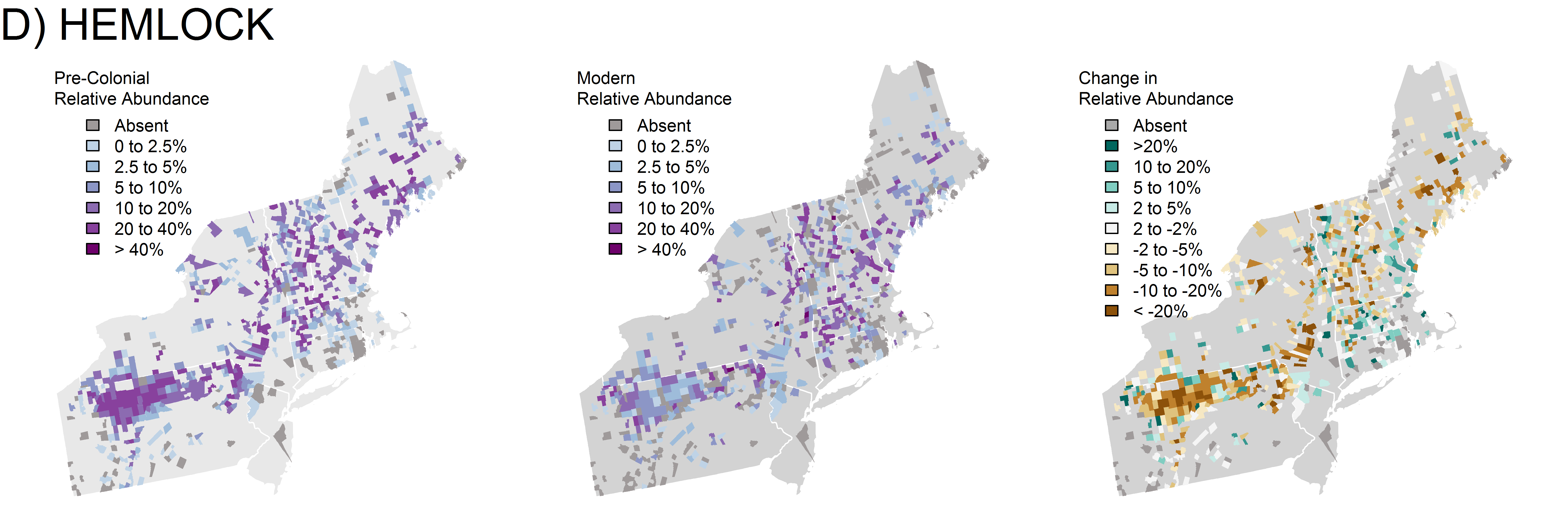

Supplement: Figure S1 — Maps of relative abundance and change for all taxa. (ZIP) [file pone.0072540.s001.zip › taxa_change_figs/HEMLCK.tif]

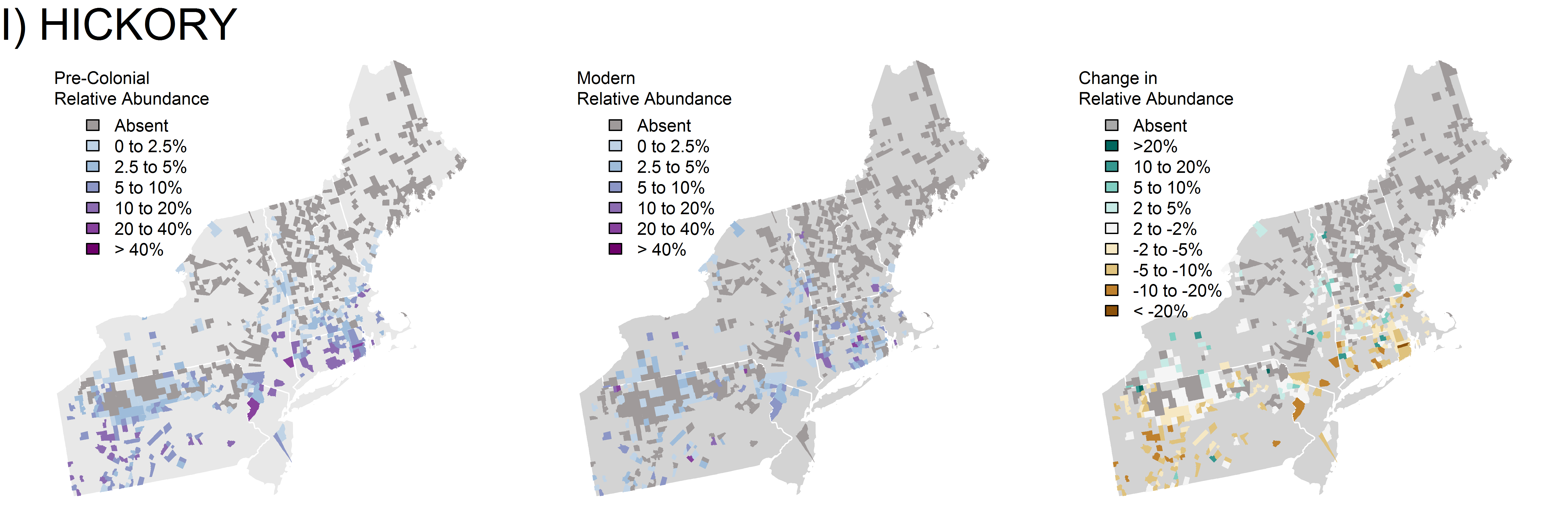

Supplement: Figure S1 — Maps of relative abundance and change for all taxa. (ZIP) [file pone.0072540.s001.zip › taxa_change_figs/HICKRY.tif]

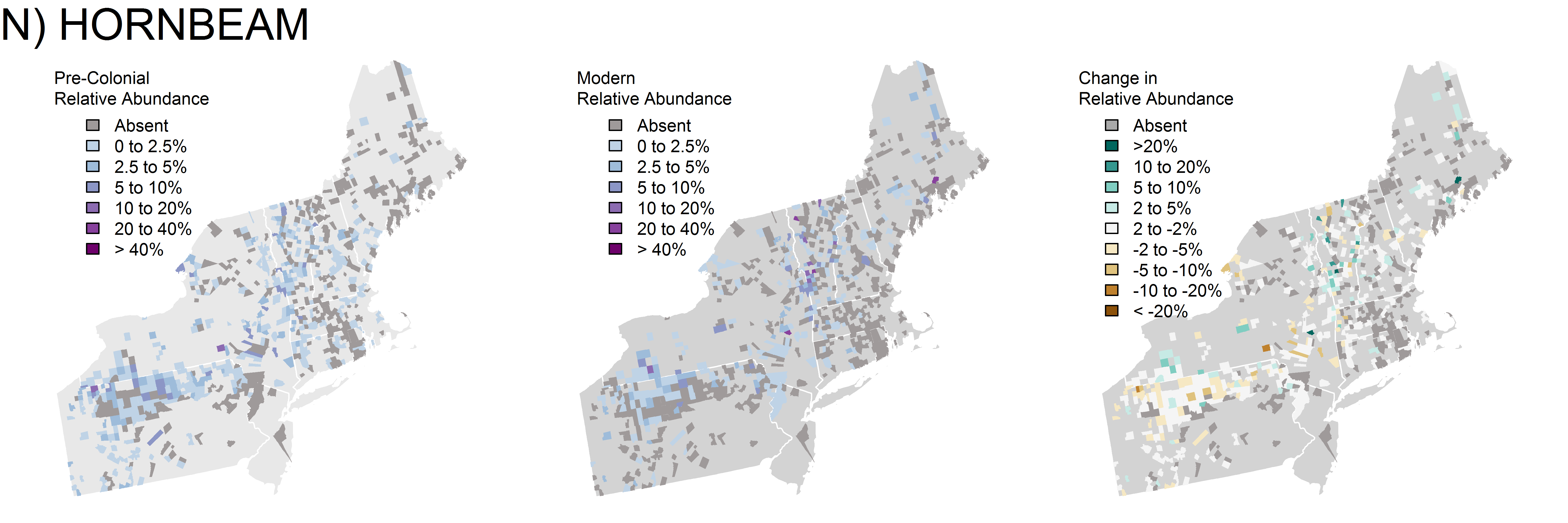

Supplement: Figure S1 — Maps of relative abundance and change for all taxa. (ZIP) [file pone.0072540.s001.zip › taxa_change_figs/HORNBM.tif]

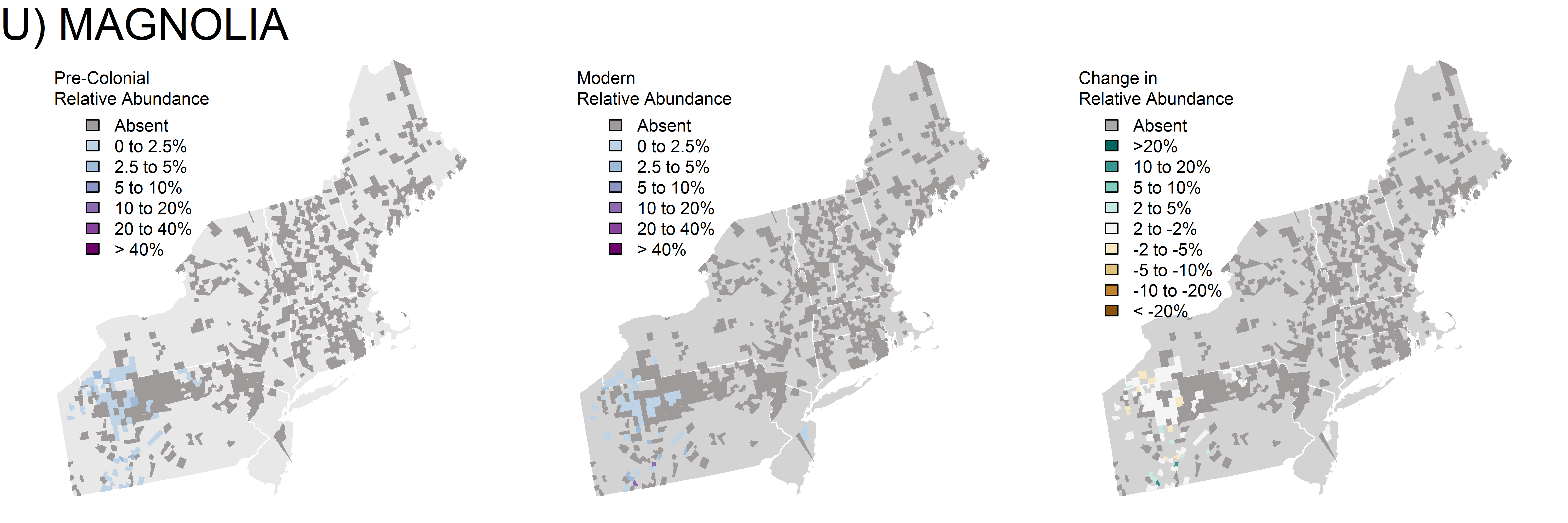

Supplement: Figure S1 — Maps of relative abundance and change for all taxa. (ZIP) [file pone.0072540.s001.zip › taxa_change_figs/MAGNOL.tif]

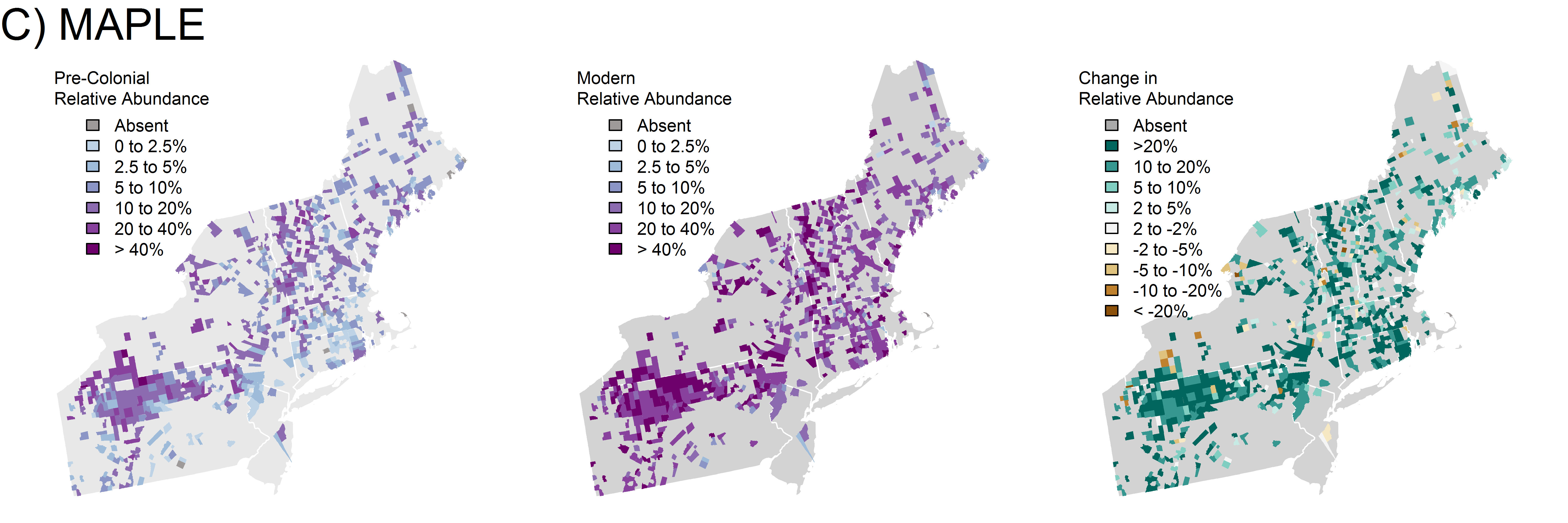

Supplement: Figure S1 — Maps of relative abundance and change for all taxa. (ZIP) [file pone.0072540.s001.zip › taxa_change_figs/MAPLES.tif]

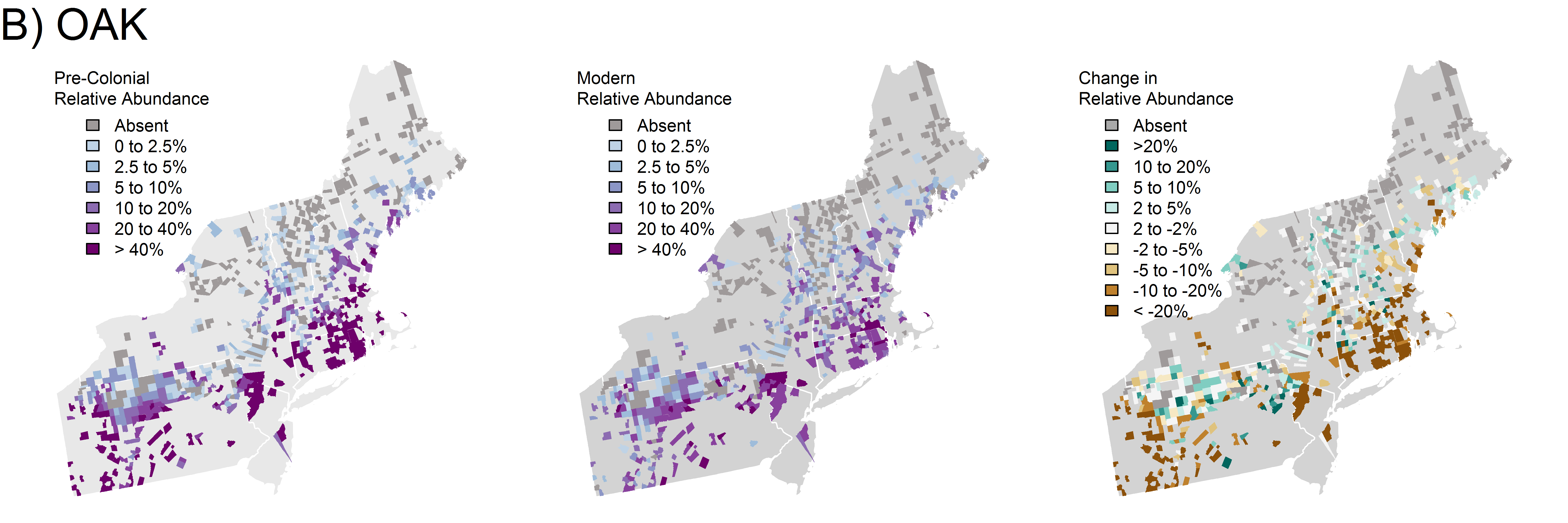

Supplement: Figure S1 — Maps of relative abundance and change for all taxa. (ZIP) [file pone.0072540.s001.zip › taxa_change_figs/OAKS.tif]

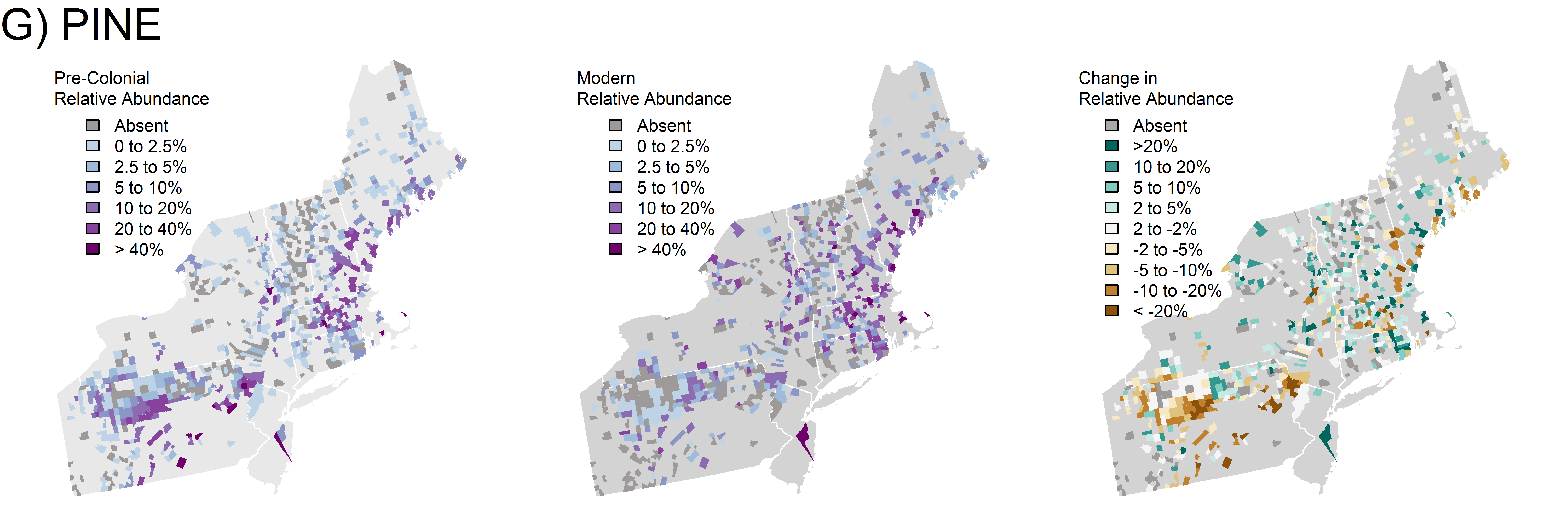

Supplement: Figure S1 — Maps of relative abundance and change for all taxa. (ZIP) [file pone.0072540.s001.zip › taxa_change_figs/PINES.tif]

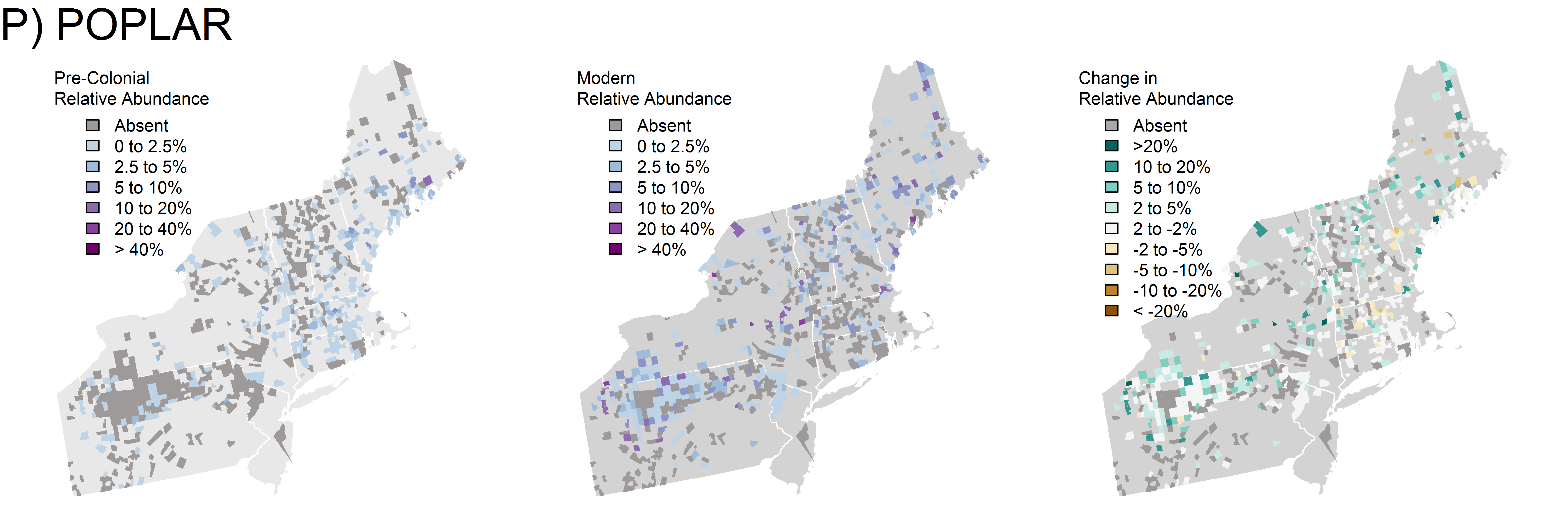

Supplement: Figure S1 — Maps of relative abundance and change for all taxa. (ZIP) [file pone.0072540.s001.zip › taxa_change_figs/POPLAR.tif]

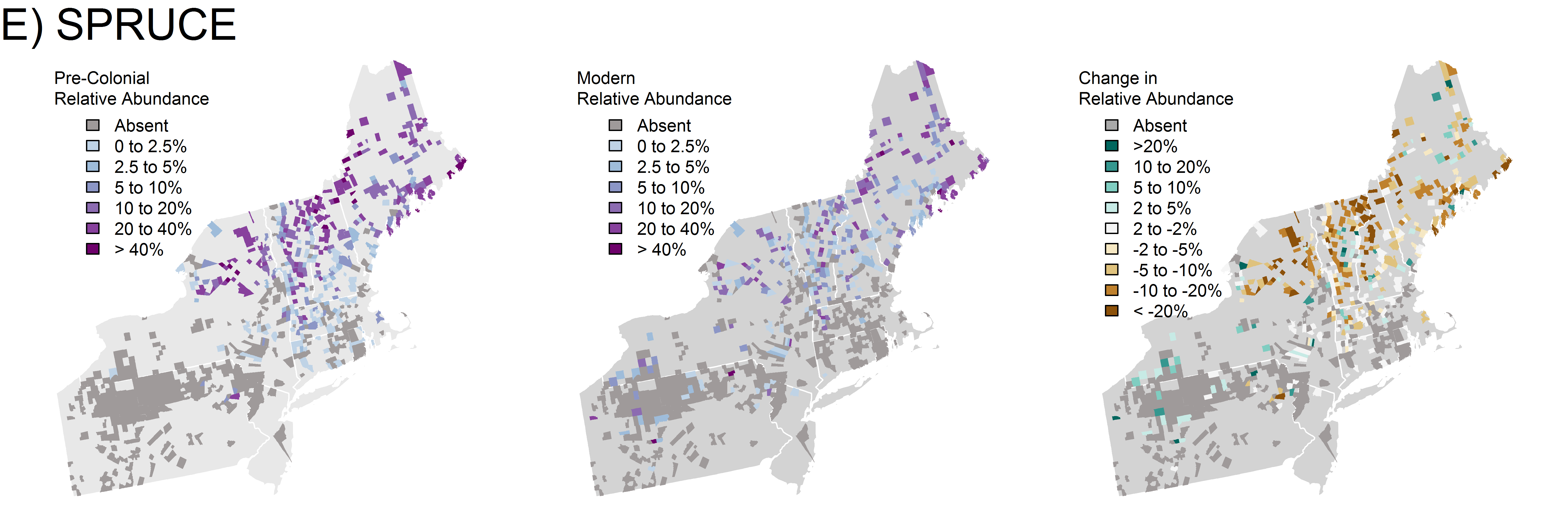

Supplement: Figure S1 — Maps of relative abundance and change for all taxa. (ZIP) [file pone.0072540.s001.zip › taxa_change_figs/SPRUCE.tif]

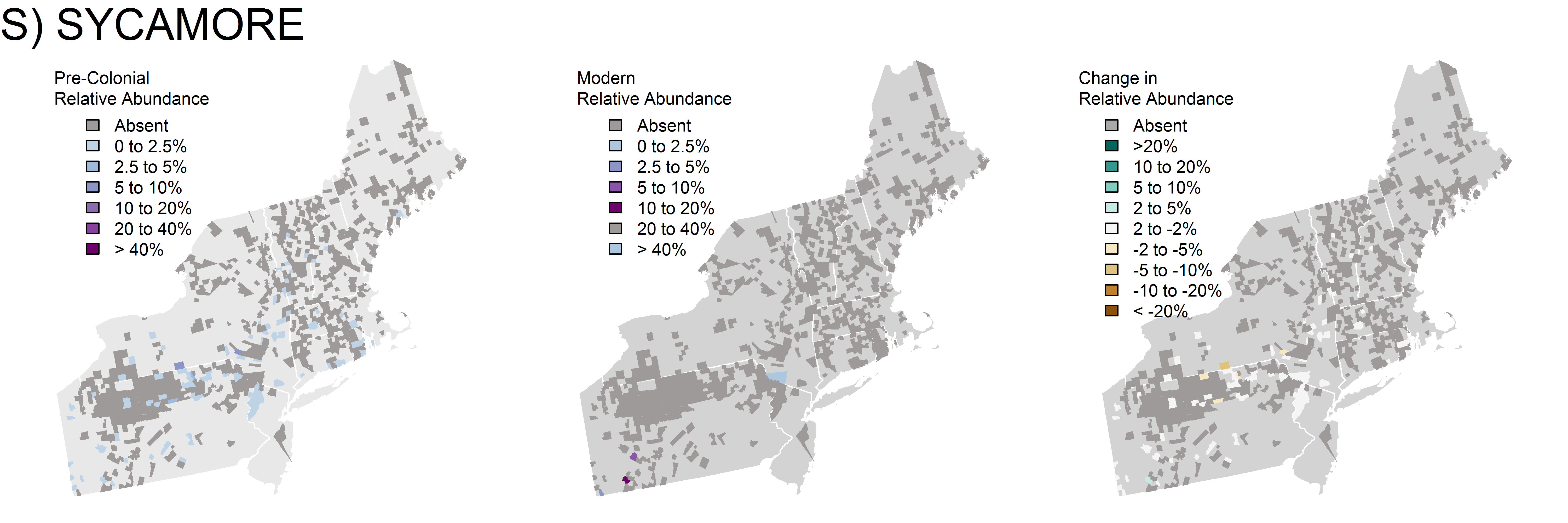

Supplement: Figure S1 — Maps of relative abundance and change for all taxa. (ZIP) [file pone.0072540.s001.zip › taxa_change_figs/SYCMOR.tif]

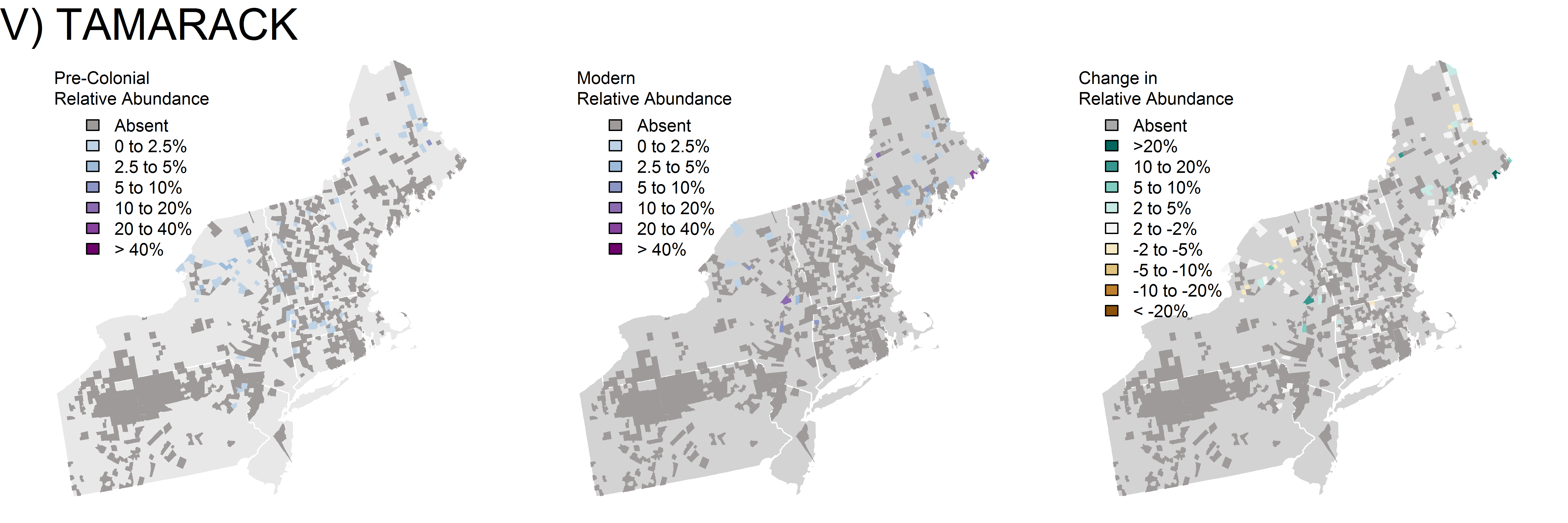

Supplement: Figure S1 — Maps of relative abundance and change for all taxa. (ZIP) [file pone.0072540.s001.zip › taxa_change_figs/TAMRAC.tif]

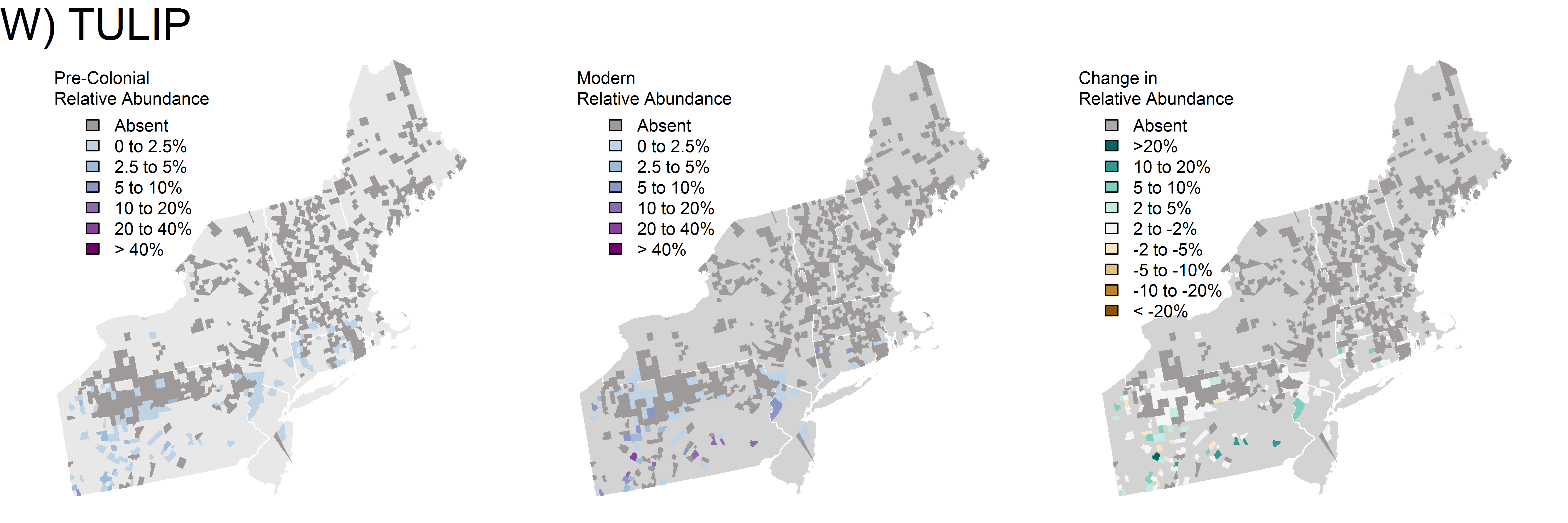

Supplement: Figure S1 — Maps of relative abundance and change for all taxa. (ZIP) [file pone.0072540.s001.zip › taxa_change_figs/TULIP.tif]

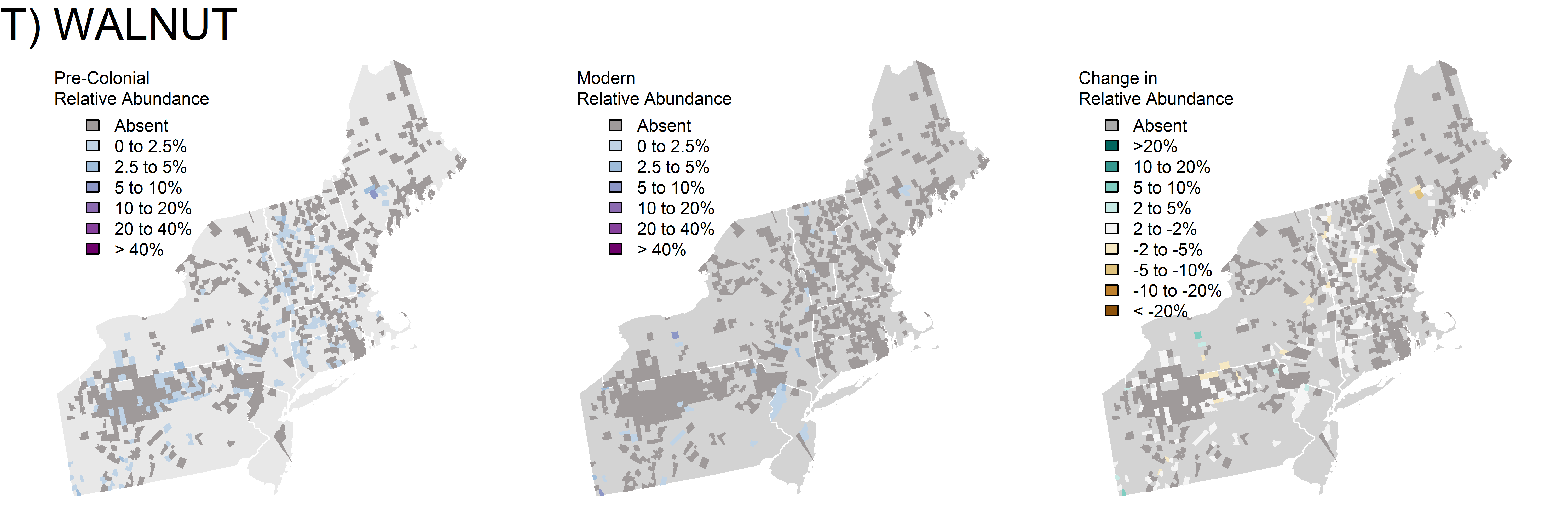

Supplement: Figure S1 — Maps of relative abundance and change for all taxa. (ZIP) [file pone.0072540.s001.zip › taxa_change_figs/WALNUT.tif]
